# Dynamics affecting the risk of silent circulation when oral polio vaccination is stopped

**DOI:** 10.1016/j.epidem.2017.02.013

**Published:** 2017-09

**Authors:** J.S. Koopman, C.J. Henry, J.H. Park, M.C. Eisenberg, E.L. Ionides, J.N. Eisenberg

**Affiliations:** a Department of Epidemiology, University of Michigan School of Public Health, United States; b Department of Statistics, University of Michigan School of Literature, Science, and the Arts, United States

**Keywords:** Polio, Transmission, Modeling, Eradication, Waning

## Abstract

Waning immunity could allow transmission of polioviruses without causing poliomyelitis by promoting silent circulation (SC). Undetected SC when oral polio vaccine (OPV) use is stopped could cause difficult to control epidemics. Little is known about waning. To develop theory about what generates SC, we modeled a range of waning patterns. We varied both OPV and wild polio virus (WPV) transmissibility, the time from beginning vaccination to reaching low polio levels, and the infection to paralysis ratio (IPR). There was longer SC when waning continued over time rather than stopping after a few years, when WPV transmissibility was higher or OPV transmissibility was lower, and when the IPR was higher. These interacted in a way that makes recent emergence of prolonged SC a possibility. As the time to reach low infection levels increased, vaccine rates needed to eliminate polio increased and a threshold was passed where prolonged low-level SC emerged. These phenomena were caused by increased contributions to the force of infection from reinfections. The resulting SC occurs at low levels that would be difficult to detect using environmental surveillance. For all waning patterns, modest levels of vaccination of adults shortened SC. Previous modeling studies may have missed these phenomena because (1) they used models with no or very short duration waning and (2) they fit models to paralytic polio case counts. Our analyses show that polio case counts cannot predict SC because nearly identical polio case count patterns can be generated by a range of waning patterns that generate different patterns of SC. We conclude that the possibility of prolonged SC is real but unquantified, that vaccinating modest fractions of adults could reduce SC risk, and that joint analysis of acute flaccid paralysis and environmental surveillance data can help assess SC risks and ensure low risks before stopping OPV.

## Highlights

• Silent circulation (SC) of wild polio viruses (WPV) when oral polio vaccine (OPV) use is stopped, could threaten eradication.
• We analyzed a model designed to develop theory about mechanisms and factors that lead to SC and how SC risks can be assessed using surveillance data.
• Prolonged low-level SC emerges as a threshold phenomenon through a mechanism related to balancing contributions of different populations to the effective reproduction number.
• Factors that promote this mechanism are many years of inadequate vaccination efforts, ongoing waning of immunity against transmission years after last OPV or WPV infection, low transmissibility of OPV, and high transmission conditions.
• Analyzing acute flaccid paralysis surveillance or environmental surveillance data by themselves cannot assess the risk that an SC threshold has been passed, but new methods to analyze them jointly could do so.

## 1 Introduction

The final stages of polio eradication are approaching. The eradication campaign began in 1988 and has lasted almost 30 years. During that time, it encountered and overcame many foreseen and unforeseen challenges. During the first couple decades of the eradication program, detecting paralytic cases through acute flaccid paralysis surveillance (AFPS) (Grassly, 2013, Heymann et al., 2004) was sufficient to direct vaccination efforts to where they were most needed. Paralytic poliomyelitis cases occur almost exclusively after first infections. Transmissions between people who have been either previously infected or successfully vaccinated are silent in the sense that they are unlikely to be detected by AFPS. Such transmission events have not to date been an obstacle to eliminating polio from most of the world. However, the few countries where elimination has yet to be achieved are unique in their conditions of high transmissibility and long periods of inadequate vaccination. These conditions could alter how long silent circulation (SC) can be sustained. SC begins with the last AFPS case detected and ends either with a new AFPS case detection or complete eradication of all infections.

Using a transmission model, we develop theory that relates SC duration to waning immunity dynamics, transmissibility of both oral polio vaccine (OPV) and wild polio virus (WPV), and vaccination rates over time. We clarify how and why these factors may extend SC beyond the current three year criteria used to define when a country is considered polio-free. The new insights we generate contribute to a theoretical framework that can help guide decisions regarding what data should be used to decide when to stop OPV use and what actions will minimize SC when OPV use is stopped.

The potential for prolonged SC is currently an issue because the final polio eradication plan requires cessation of all oral polio vaccine (OPV) use (WHO, 2015a). The need to cease OPV use arises because the vaccine strain can cause paralysis and can be transmitted and evolve to cause paralysis and to transmit as readily as wild polio viruses (WPV) (Burns et al., 2014, Famulare et al., 2015). OPV cessation must be coordinated worldwide because of the risks that introduction of OPV from an area using it to an area not using it will lead to sustained OPV transmission and evolution into circulating vaccine derived polio viruses (cVDPV) that can cause epidemic polio is great (Famulare et al., 2016, WHO, 2015a, WHO, 2015c). Inactivated polio vaccine (IPV) will replace OPV. But IPV does not stimulate immunity against infection and transmission in children who have never experienced an OPV or WPV infection (O’Ryan et al., 2015, Piirainen et al., 1999). Thus, after OPV cessation children vaccinated only with IPV will rapidly accumulate and amplify any transmission that exists (Famulare et al., 2016). If sufficient OPV is available, the setback to eradication from undetected SC after OPV cessation may be manageable even though localized reinstitution of OPV vaccination runs a high risk of generating circulating vaccine derived epidemics (Famulare et al., 2016). But the intention is to eradicate all strains of OPV because if released they could evolve into as much of a problem as WPV (Previsani et al., 2015, WHO, 2015b). That means destroying all OPV vaccine reserves. If SC persisted up to the point when OPV was destroyed, control potential would revert to where it was before Sabin developed OPV.

Action is therefore required to insure the chances of prolonged SC are minimal. Two different surveillance systems are being used to this end. AFPS has been highly developed during eradication and is the major surveillance system that has helped eradication efforts reduce transmission to only three countries (Grassly, 2013, Heymann et al., 2004). All cases of acute flaccid paralysis under age 15 are cultured for polio virus. Globally AFPS has been designed to cover every population anywhere in the world with risks of polio transmission. In crucial populations, not only AFP cases are cultured, but their contacts are cultured as well. A newer system, environmental surveillance (ES), involves collecting samples from sewage systems or other feces contaminated black waters and identifying and sequencing polioviruses (Alam et al., 2016, Asghar et al., 2014, Hovi et al., 2012). It indicates the presence of virus in a population but does not identify source cases. It is not being designed as a comprehensive system covering all populations.

A relevant question addressed in this paper is where ES should be established to best detect SC. Answering that question requires an understanding of what generates SC that this paper provides.

SC has two major potential causes. The cause on which the eradication effort has focused to date is inadequate AFPS. We focus in this paper on elucidating a second potential cause of SC. It is waning immunity that increases the potential for individuals with either OPV or WPV induced immunity to become reinfected and transmit infection.

To pursue a better understanding of SC causes, we pursue a better theoretical framework for assessing the effects of waning immunity on SC. To this end we analyzed a simple model as a first step in inference robustness assessment (Koopman et al., 2016). We simplified the polio model we developed to examine success and failures of the polio eradication effort in the context of waning immunity and OPV transmissibility (Mayer et al., 2013). Model simplifications allowed us to see clearly the contributions of different groups and different parameters to the effective reproduction number. The theoretical framework this allowed us to develop has the potential to extract considerable information about waning from combined AFPS and ES data gathered during recent years. We do not have access to that data. But we hope that the understanding that we generate here will increase the capacities for risk assessment by those who do.

The major data used in modeling efforts to date are AFPS and vaccination coverage data. But we uncover an identifiability problem of AFPS data for predicting SC if there is waning immunity. We do this by illustrating how models that do and models that do not generate SC can be fitted to the same AFPS data. Immunity against paralytic polio is thought not to wane. But immunity against transmission clearly does (Abbink et al., 2005, Grassly et al., 2012, Jafari et al., 2014). A recent analysis of more direct data on waning of immunity has succeeded in developing a new theoretical framework for modeling waning immunity (Famulare et al., 2016). Waning formulations in that paper are realistic and supported by data. But our simpler approach elucidates issues that all more realistic models must address but are hard to see in them.

Our analysis seeks to uncover how and why the following interact to affect SC risks: (1) waning immunity patterns, (2) high transmission potential, and (3) delays in getting vaccination to levels needed to reduce cases to very low levels. We also seek to develop theory to predict how vaccine transmissibility and infection to paralysis ratios (IPR) affect SC. Additionally, we explore how vaccination of age groups with waned immunity before stopping OPV could help insure eradication given different waning patterns.

## 2 Methods

### 2.1 Infection transmission model

**Fig. 1:**
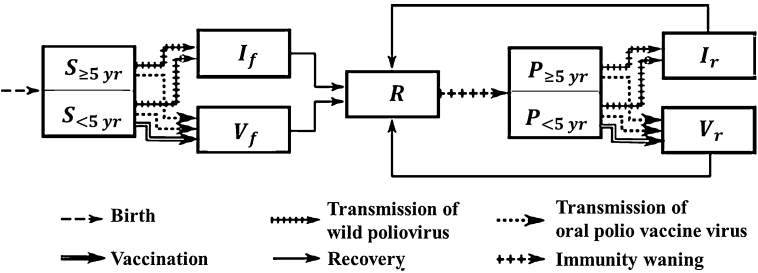
The seven compartments in our one step waning model and its flows not related to age or death are illustrated. The one waning step is from R to P. The speed of that flow is the speed of waning. The depth of waning is determined by the degree of susceptibility in the P compartment and the duration and contagiousness of the WPV or OPV infections that flow out of P. Although we distinguish between different age groups in all seven compartments (in order to allow for realistic aging), we only show here the distinctions that are relevant to infection processes, namely between (fully or partially) susceptible individuals who are or are not in the age group targeted by vaccination efforts.

Our transmission model is illustrated by the flow diagram in Fig. 1. It distinguishes first infections from subsequent reinfections and WPV infections from OPV infections. Waning in the model occurs in a one-step transition from recovered state R to a partially susceptible state P. Recovery from all infections leads to a single immune state R. That means immunity from WPV or OPV infections is identical, as is immunity resulting from a first infection or a subsequent infection.

#### 2.1.1 Simplifying model assumptions

Our model analyzes only one polio serotype at a time. It uses parameters such as the fraction of first infections that result in paralytic poliomyelitis and the relative transmissibility of OPV compared to WPV, that have been used by others (Duintjer Tebbens et al., 2013c) to characterize the three serotypes of polio. It assumes homogeneous mixing and homogeneous contact rates across all compartments with paralytic cases transmitting identically to non-paralytic first infection cases. It assumes that death rates are constant, not affected by age, and are equal to birth rates. The differential equation model form used essentially assumes a continuous population rather than discrete individuals. It also assumes everyone in the population has an equal rate of contacting everyone else. Model equations and parameters are presented in Appendix.

The model does not directly distinguish paralytic infections from silent infections. But our analyses of model output use the first infections to paralysis ratio (IPR). When the cumulative number of first infections in the past year becomes less than the IPR, the time of the last paralytic polio case is declared. A new count of first infections then begins and goes on without time limit. When that total count exceeds the IPR, a time of reappearance or recurrence of polio cases is declared. We assume that reinfections do not cause paralytic poliomyelitis.

Eradication is said to occur in our model when the prevalence of infection falls below 1 in a population of one million. We explored a variety of definitions that counted different entities such as numbers of first and repeat infections weighted by their overall transmission potential. We found that none of these affected the basic conclusions of this paper.

Different duration and contagiousness of infection are assigned to wild polio virus (WPV) and oral polio vaccine virus (OPV) infections. We assume that OPV infections have the same duration and contagiousness whether they arose from direct vaccination or transmission from someone with an OPV infection. To further reduce parameters, we assume that ratios affecting relationships between first and reinfections are the same for OPV and WPV infections and that the ratios affecting duration and contagiousness of OPV compared to WPV infections are the same for first infections and reinfections.

#### 2.1.2 Age structure

To examine vaccination effects, we add age to the model because vaccination is only given to children less than 5 years old. Instead of making chronological time and aging time move across the same small time steps, we reduce the computations needed for model analysis by moving age progression across larger steps (1.5 months) than chronological time and keeping track of age only until age 5. We confirmed that this does not cause inappropriate leakage of susceptibles out of the vaccine ages to older ages.

#### 2.1.3 Modeling vaccination patterns over time

Patterns of vaccination vary greatly in the real world and extensive modeling work has demonstrated how important capturing the past pattern of vaccinations accurately is to making valid predictions of current and future risks that arise from inadequate vaccination programs in the past (Duintjer Tebbens et al., 2014, Duintjer Tebbens et al., 2013b, Duintjer Tebbens et al., 2013c, Duintjer Tebbens et al., 2013d, Duintjer Tebbens et al., 2015, Duintjer Tebbens and Thompson, 2014, Duintjer Tebbens and Thompson, 2015, Pons-Salort et al., 2016, Thompson, 2013, Thompson and Duintjer Tebbens, 2014a, Thompson and Duintjer Tebbens, 2014b; Thompson et al., 2015b, Thompson et al., 2015a Thompson et al., 2013a, Thompson et al., 2013b, Thompson et al., 2013c; Thompson and Tebbens, 2012). But we want to show why these models will not adequately capture the risk of prolonged SC. So, we use a generic vaccination pattern that can do that.

Vaccination patterns over time were simplified by assuming all children were vaccinated at the same rate at any moment in time. Rather than fitting vaccination patterns to any one country, we characterized a general pattern found in the places where polio held on the longest. That pattern involved periods of more intense vaccination that knocked down transmission followed by less intensification of efforts or even diminution of efforts after initial success. This is followed by boosted vaccination levels that finally eliminate polio cases. The characteristic of the real world that our model of vaccination patterns sought to capture was variability in the time that WPV transmission was knocked down without being knocked out before a final boost in vaccination levels leads to elimination of polio cases. We used a linear increase in vaccination during the delay time before the final boost. When there is a delay, we begin at zero and linearly ramp up vaccinations across the delay interval to a level that achieves a first infection prevalence of 300 at the end of the ramp. That vaccination level varies by the length of vaccine ramp-up, the type of waning, and the relative transmissibility of the vaccine. It is determined by fitting the vaccination level to the prevalence level at the end of the vaccination ramp up. Using this tactic allows us to vary the time that vaccination knocks transmission down without knocking it out while keeping conditions at the time of a boost in vaccination constant. In appendix section 5, we examine how using values lower than a prevalence of 300 affects system dynamics and the inferences made in the main paper. Examples of vaccination patterns are found in Fig. 5, Fig. 6, Fig. 7. For model settings where there is no delay, the rate of vaccination is constant at its final level from the onset of vaccination.

#### 2.1.4 Waning model rationale and description

Given the complexity of immune control of infection, waning immunity potentially has many dimensions. We choose to reduce this complexity to a single flow from the recovered (R) stage to the partially susceptible stage (P). Two parameters determine the waning process: the waning rate, and the waning depth or the fraction of transmission potential (basic reproduction number) that a population of all individuals in the P state would have compared to a population of individuals all in S. The waning depth has three components that we set to equal values. These are the relative susceptibility of individuals in the P compartment with respect to those in the S compartment, the relative contagiousness per unit time of reinfections with respect to first infections, and the relative duration of reinfections with respect to first infections. Because transmissibility is the product of contagiousness and duration, transmissibility wanes more than susceptibility. The waning depth is the triple product of the fraction of waning in susceptibility, the fraction of waning of contagiousness, and the fraction of waning of duration.

**Fig. 2:**
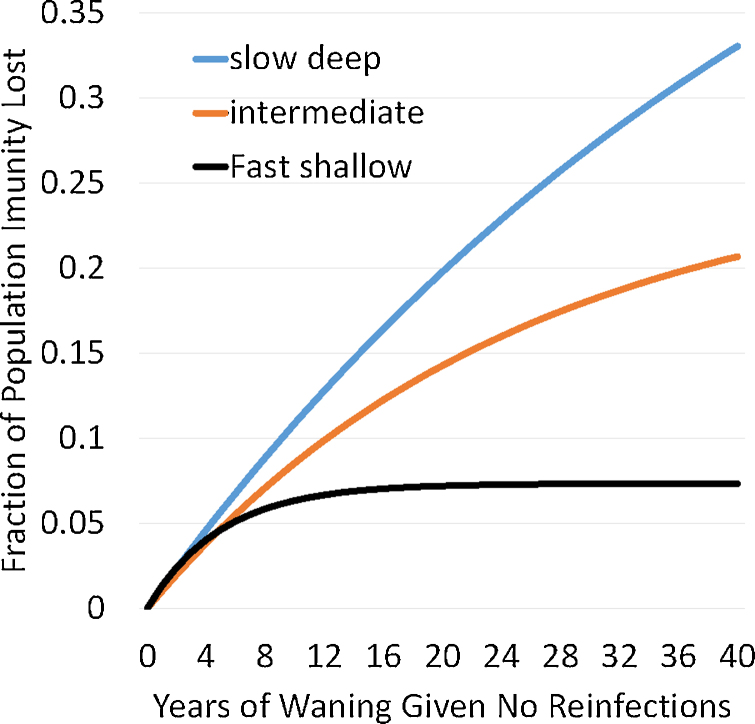
Population extent of waning for the different waning scenarios as defined by waning depth affecting susceptibility times waning depth affecting duration, times waning depth affecting contagiousness under the assumption that all three effects have the same waning depth parameters. Waning rate and depth parameters used are from Table 1.

To demonstrate the effect of different waning patterns, we chose three illustrative values of the waning rate. We then found three corresponding values for the waning depth which, in the absence of vaccination, generate the same exponential distribution of ages of infection in each pair of waning rate and depth parameters. We used pair values that give a slow-deep waning pattern, a fast-shallow waning pattern and an intermediate waning pattern as illustrated in Fig. 2 and specified in Table 1. Note that the quantity shown in Fig. 2 is the product of four elements (1) the fraction of the population that would be in P state if everyone started in the R state and never experienced any subsequent infection, (2) the susceptibility of someone in the P state divided by that of someone in the S state, (3) the contagiousness of someone in the P state who gets infected divided by that of someone who got infected from the S state, and (4) the duration of infection for infections from the P state divided by duration of infections from the S state. All four of these elements determine effective reproduction numbers as shown in Appendix. In Appendix section 3 we analyze at equilibrium how model parameters affect the ratio between transmissions from first infections and reinfections.

**Table 1:** Transmission, vaccination, and waning parameters. This analysis is performed using type 3 polio parameters for the paralysis to infection ratio of 1000 and the relative transmissibility of OPV to WPV of 0.37. The first three columns of numbers are set parameter values with the caveat that the waning depths for B–D were selected to give approximately the same *R*_0_ values. Then those three values were used for waning depths F–H. The basic reproduction number is set to the value found to give the desired average age at first infection at equilibrium before vaccination is begun. The effective contact rates at these settings are indicated in the last column. The vaccination rates are those that give a prevalence of first infections of 300 per million at the end of the ramp up period and are found separately for each of the eight settings given the values in the first four columns.

Waning scenarios in Fig. 4	Panel	Avg. age 1st Inf no vaccine	Waning rate	Waning depth	Basic *R*_0_	Vaccine rate at end of 20 yr ramp up	Effective contact rate
No waning higher transmission	A	3.33	0	—	15	0.25	195.3
Fast shallow higher transmission	B	3.33	0.2	0.073	9.3	0.33	121.5
Intermediate higher transmission	C	3.33	0.04	0.26	9.3	0.42	121.5
Slow deep higher transmission	D	3.33	0.02	0.6	9.3	0.45	121.5
No waning lower transmission	E	5	0	—	10	0.20	130.2
Fast shallow lower transmission	F	5	0.2	0.073	6.9	0.25	89.8
Intermediate lower transmission	G	5	0.04	0.26	6.5	0.31	83.4
Slow deep lower transmission	H	5	0.02	0.6	6.4	0.35	83.3

The average age of infection in our model is determined jointly by the basic reproduction number and by waning parameters since the first generates a force of infection from first infections and the later generates a force of infection from reinfections. We examined two different average ages of infection at equilibrium before vaccination was begun: Five years for lower transmission and 3.333 years for higher transmission. The age distribution at time of first infection for all of our high transmission settings result in an *R*_0_ estimate of 15 using the methods of (Fine and Carneiro, 1999); our low transmission settings would likewise all result in an *R*_0_ estimate of 10. These two values represent high and low values for “poor developing countries” in (Fine and Carneiro, 1999) Fig. 1. The basic *R*_0_ in Table 1 is the number of infections that a single first time infected individual would generate if all other individuals in the population were in the completely susceptible S state. How we set parameter values is in the legend of Table 1. That the values in Table 1 generate dramatically less waning than the model by the Institute for Disease Modeling (Behrend et al., 2014, Wagner et al., 2014) is illustrated in Fig. 7 of the fourth Appendix section.

Our primary outcome variables of interest are duration of silent circulation and outcome of silent circulation. Silent circulation duration is defined in the model output from the time that cumulative incidence of first infections over the past year falls below the first infection to paralysis ratio (IPR) until either the cumulative incidence of first infection rises once again above the IPR (recurrence) or the prevalence falls below an average of one individual in the population (eradication). Stable eradication at a given level of vaccination occurs when the equilibrium effective reproduction number from both first infections and reinfections is below one. The reproduction number may continue to rise for many years after the last polio case when there is slow or intermediate waning. Stability, therefore, is determined by running models for a long time. Given stable eradication, reintroduction of infection from the outside after local transmission has ceased will never result in an epidemic. With unstable eradication, however, individuals with waned immunity in the P state from Fig. 1 will eventually put the effective reproduction number above one. True global eradication that eliminates all virus in people and laboratories would be practically stable but could be mathematically unstable.

### 2.2 Model equations and numerical solutions

The model equations are presented in the appendix along with the code used to implement those equations in Berkeley Madonna (Oster and Macey, 2015).

### 2.3 Model analysis

The model is analyzed both mathematically and numerically. Numerically the model is run to WPV equilibrium before introducing OPV vaccination. Numerical analyses were checked to see that they were not affected by the numerical integration algorithms used or the time steps employed. Mathematical analysis is presented in Sections 2–4 of the Appendix.

## 3 Results

### 3.1 Model generated patterns of drop in first infection prevalence

**Fig. 3:**
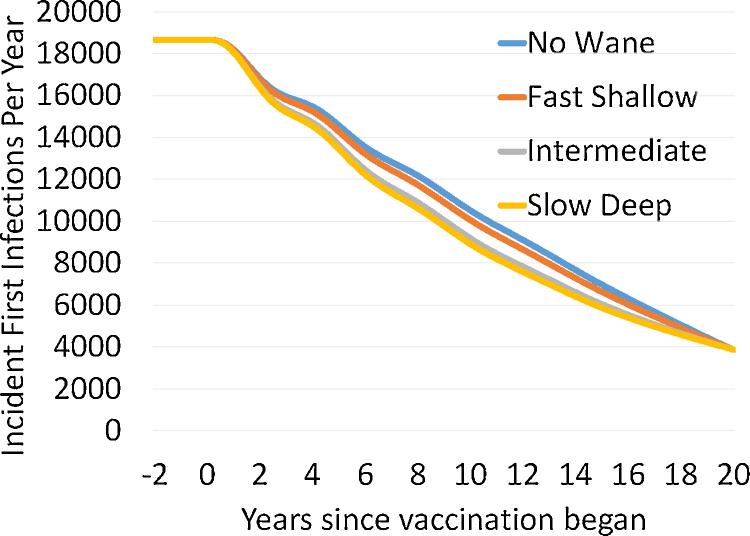
First infection incidence as a function of waning scenario across a 20 year delay period during which first infection prevalence is reduced to 300 per million for the higher level of transmission. Settings used are those for a type 3 virus and settings A–D from Table 1.

Fig. 3 shows the pattern of decreasing incidence of first infections during a 20 year ramp up delay of vaccination for parameters in Table 1. Given the infection to paralysis ratio of 1000 used in this analysis and the prevalence of first infections of 300 at the end of the ramp up, 3–4 polio cases per year in our population of one million would be expected at the end of the ramp up. The consequences of using first infection prevalence values at the end of the vaccination ramp up period other than 300 cases are presented in Appendix section 5.

The procedure for setting parameters outlined in Table 1 legend was chosen to make the force of infection identical at both the endemic equilibrium and at the end of the ramp up, for all waning scenarios. By doing this, we demonstrate that the pattern of polio cases over time in a population cannot be used to determine the waning pattern in that population. That inference is important because in the next section we will show that waning patterns with similar effects on the population level of infection before vaccination is begun have large effects on silent circulation duration after the last polio case. Note that there is a tradeoff between settings for waning parameters and Basic *R*_0_ (or the contact rate used to set that) and that we chose values for the set waning rates and depths that kept the average age at first infection constant for the higher transmission settings given that there was any waning at all. That multi-parameter tradeoff makes the lack of identifiability illustrated in Fig. 3 even greater. The way we present Fig. 3 emphasizes the role of effective vaccination rates as a single factor affecting identifiability. If effective contact rates, average age of first infection in the pre-vaccine era, and infection to paralysis parameters were known, adding information about effective vaccination levels might theoretically help determine waning patterns. From Table 1 we see there is some increase in vaccination level needed to get to our fixed prevalence of first infections as we progress through fast-shallow to slow deep waning scenarios. Data and modeling of vaccination rates has greatly increased in recent years (Pons-Salort et al., 2016). But given the difficulties in determining what the effective levels of vaccination have been in populations over longer times, the waning patterns will not be identifiable from standard polio program data.

### 3.2 Silent circulation patterns

SC begins in our model when vaccination brings down cumulative incidence of first infections over the past year to less than the first infection to paralysis ratio. That point is a likely time when the last polio case might be detected. SC ends with either mathematically stable eradication, mathematically unstable eradication, or recurrence of polio cases. We declare eradication when the prevalence of infection goes below one. If the cumulative incidence of first infections since the last polio case rises above the IPR before the prevalence goes below one, then we declare a recurrence of infection.

**Fig. 4:**
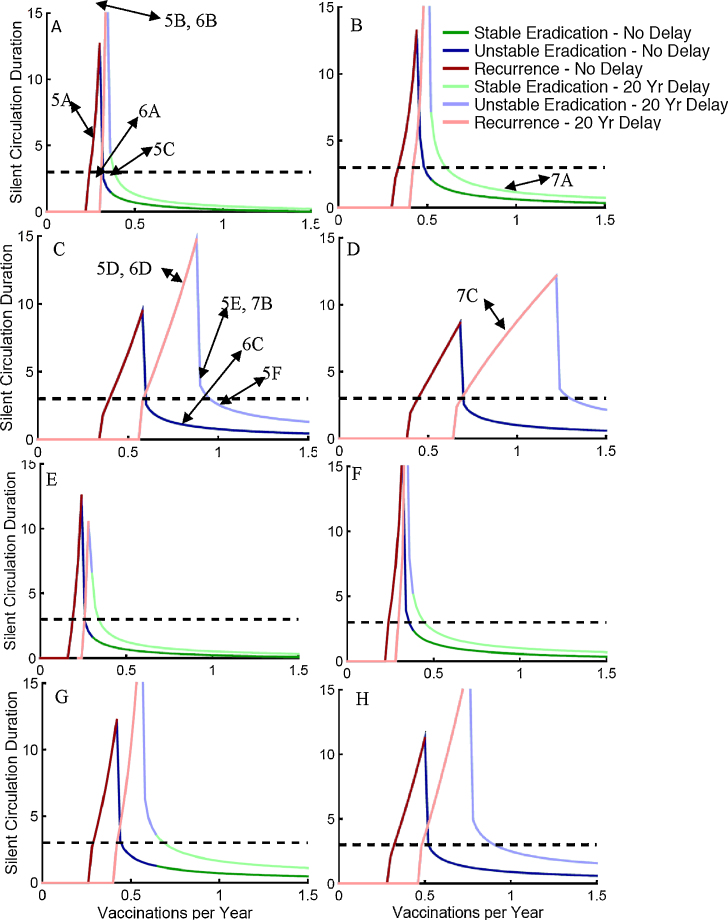
Patterns of duration of silent circulation as a function of final total vaccination rates in less than five year olds by waning immunity scenarios: (A and E) no waning, (B and F) fast shallow waning; (C and G) intermediate waning; and (D and H) slow deep waning. (A–D) have an average age of first infection of 3.33 years while E–H have an average age of 5. All panels have type 3 settings (IPR = 1000, OPV/WPV transmission ratio = 0.25). Type of silent circulation (stable, unstable, and recurrent) is indicated by color. Horizontal dashed line illustrates 3-year silent circulation. Parameter values used are shown in Table 1. The points on these curves where the dynamics are illustrated in subsequent figures are indicated by figure number and panel letter of Figs. 5–7.

Fig. 4 presents SC duration by the final stable rate of effective vaccination of children under age 5. This is lower than the actual vaccination rate. It is the rate at which vaccine infection occurred and stimulated immunity in completely susceptible children. Two patterns of vaccination are presented. In the first there is no delay in vaccination reaching its final levels. In the second there is a 20-year delay during which vaccination is linearly ramped up to a level that generates a first infection prevalence of 300 per million total population. The prevalence paths taken to this value are indicated in Fig. 3. Parameters used are indicated in Table 1. Whether the SC ended with a recurrent polio case, unstable eradication where a new introduction could lead to a new outbreak, or stable eradication where outbreaks would be small is indicated by color for different vaccination levels. The no delay curves always occur at lower vaccination levels than the 20-year delay curves.

**Fig. 5:**
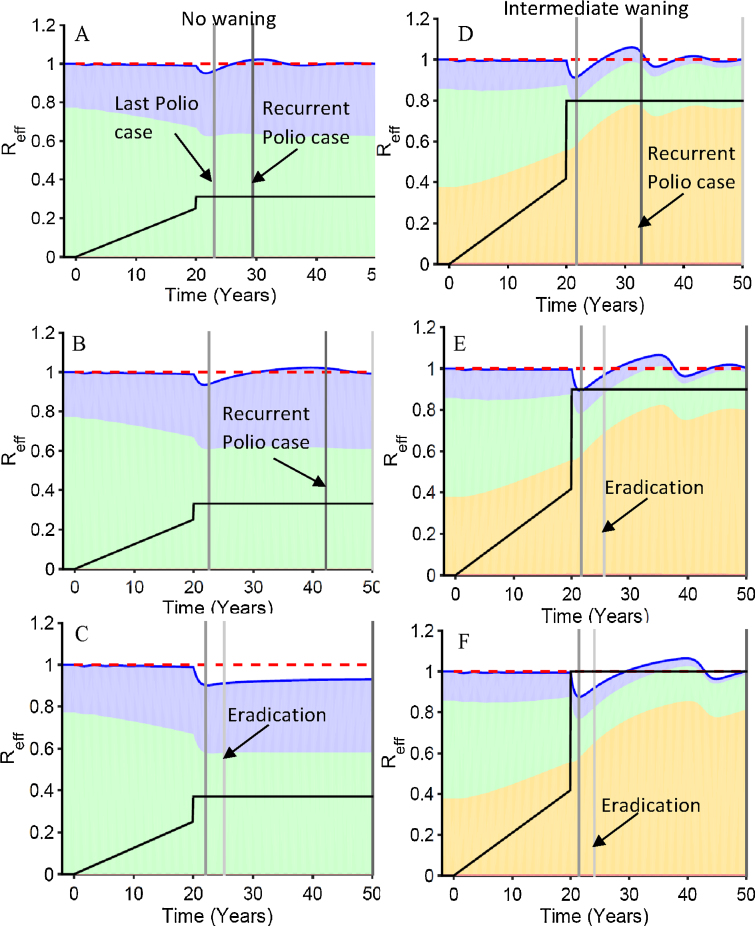
The dynamics of the effective reproduction number and its components as vaccination levels are increased. Intermediate waning, the higher level of transmission, a 20-year delay and serotype 3 settings were used. The position of each panel in Fig. 4 curves are indicated in Fig. 4. The final vaccination levels for panels are A = 0.32 effective vaccinations per year, B = 0.34, C = 0.38, D = 0.8, E = 0.9, F = 1.0. The black vaccination level lines ramping up to 20 years and then jumping to the final vaccination level use the same left hand scale as the effective reproduction numbers. The blue shaded area is the contribution to the effective reproduction number from first infections in the five and older age group, the green is first infections from the under 5, the yellow is reinfections in the five and older, and the red (barely visible below the yellow) is from reinfections in under age five. The dark blue line summing all shaded areas is the effective reproduction number. The red dotted line is the transmission threshold. The time of eradication, last polio case, and a recurrent polio case have increasingly darker gray shading.

**Fig. 6:**
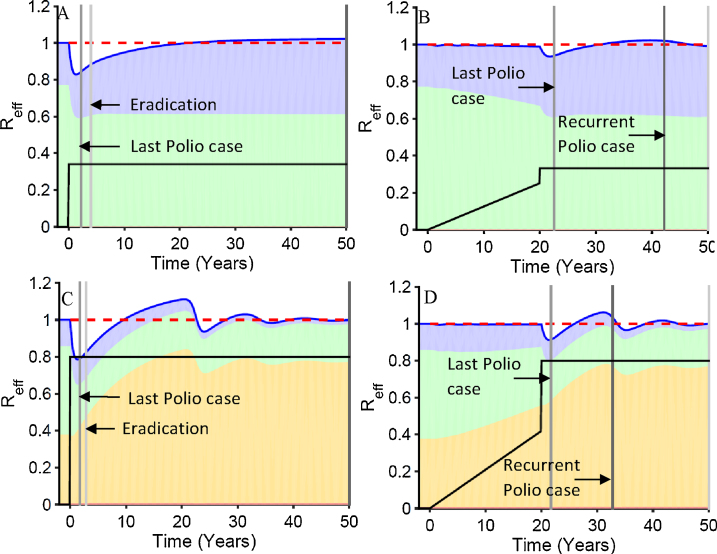
The dynamics of the effective reproduction number and its components with and without a delay in reaching final vaccination levels. Intermediate waning, the higher level of transmission, and serotype 3 settings were used. The positions of each panel on silent circulation duration curves are indicated in Fig. 4. The shading and lines have the same meaning as Fig. 5. (For interpretation of the references to color in this figure legend, the reader is referred to the web version of the article.)

**Fig. 7:**
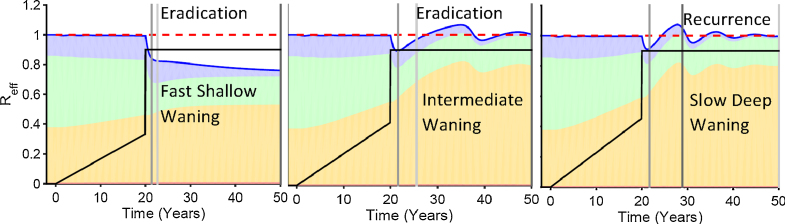
Changing dynamics of the elements of the total effective reproduction number across different waning scenarios explain the effects of those waning scenarios when final total vaccination levels are kept constant. Higher transmission parameters along with IPR and OPV/WPV transmissibility characteristic of type 3 were used.

To help understand where the points in Fig. 4 come from, let us look at Fig. 5, Fig. 6, Fig. 7. Then we will return to discuss what the patterns in Fig. 4 indicate. Rather than presenting levels of infection, Fig. 5, Fig. 6, Fig. 7 present the contributions of first infections and reinfections in the populations under or over age five to the total effective reproduction number. We made our model simple enough so that the contributions of each group of infections sum to the total effective reproduction number. So let us first explain those contributions.

Fig. 4 reveals 3 effects as waning progresses from no waning to slow deep waning across panels A–D and E–H. As waning becomes slower and deeper while keeping the effects of waning on age at first infection constant before vaccination begins, (1) it takes higher final vaccination rates for eradication to be achieved. (2) The vaccination rates required for eradication increase between situations where final vaccination rates are instantly achieved or have a 20-year delay. (3) The breadth of vaccination levels that result in prolonged silent circulation increases.

These differences are greater when transmissibility is at the upper end of the plausible range for higher transmission countries (A–D) than when it is at the lower end of that range (E–H). The differences between no waning and fast shallow waning are small. In these scenarios, there is no meaningful ongoing waning after 5 years, so that there is no ongoing accumulation of people susceptible to reinfection after 5 years. As seen in Fig. 2, there is such an ongoing accumulation of partially susceptibles with intermediate waning, and there is twice as much accumulation with slow-deep waning.

### 3.3 Effective reproduction number analyses to explain silent circulation patterns

The total effective reproduction number for our model is the sum of the effective reproduction number for first infections plus the effective reproduction number for reinfections in each of the age groups. The mathematical logic supporting this is shown in Appendix section 2.

$$R_{\text{EffTot}}=R_{\text{EffFirst}<5}+R_{\text{EffSubsequent}<5}+R_{\text{EffFirst}>=5}+R_{\text{EffSubsequent}>=5}$$

When *R*_EffTot_ is less than one, infection levels will be falling. When it is greater than one, they will be rising. The dynamic pattern of these four components helps determine whether a given vaccination level will result in stable eradication, unstable eradication, or a recurrent case of polio. How far vaccination drives the total effective reproduction number below one and how long it stays there determines how far infection levels will drop. The effective reproduction number when vaccination is first begun always starts out at the value of one in all the dynamics panels in Fig. 5, Fig. 6, Fig. 7 because we start vaccination only when the system is at equilibrium.

### 3.4 Temporal dynamics leading to the summary points in Fig. 4

Fig. 5, Fig. 6, Fig. 7 color the four contributions to *R*_EffTot_. Green is the contribution from first infections the under-five age group. Blue is first infections in the over-five group. Yellow is the reinfections in the over-five age group. Red is reinfections in the under-five age group. The contributions of this latter group are so small that they are not readily perceptible in the figures. The time when the last case is seen is indicated by the earliest vertical line. The time after that when a recurrent case is detected (when the sum of new first infections since the last case exceeds the IPR) is indicated by a darker vertical line. The time when eradication occurs (the sum of first infection in the past year becomes less than the IPR) is indicated by a lighter vertical line. The temporal patterns of vaccination levels are indicated on these figures using the same scale as the effective reproduction number.

We do not stop all transmission in these figures when the number of total infections, including first infections and reinfections, goes below one and we declare eradication. Of course, once there is eradication, in the absence of reintroductions the effective reproduction goes to zero because there are no infections. But for didactic reasons we follow dynamics beyond this point by allowing a fraction of an individual to continue transmitting. This helps us see whether eradication is stable or unstable. If the effective reproduction number ever goes back above one after eradication, the eradication is unstable and introduction of a new case could set off a large epidemic.

Note that in Fig. 5 before and during the 20 year ramp up, all panels are identical within the two waning scenarios examined (A–C no waning and D–F intermediate waning). During the 20 years of vaccination ramp up, the total effective reproduction number dips only slightly below one. But it dips enough down to drive the endemic prevalence of first infections to 300. For the no waning scenario in panels A–C of Fig. 5 there are only first infection events so the above equation has only two elements rather than four because with no waning, there are no reinfections. During the ramp up delay, vaccination reduces the contribution of first infections in the under-five age group. But that drop is compensated for by more children being susceptible at their fifth birthday so that the greater than five-year-old susceptible group contributes more to the effective reproduction number. When there is no waning, it takes only a small boost in vaccination levels to lower the effective reproduction number to where cases are no longer detected (Panel A). A very slight increase in the final vaccination level can extend the SC interval between the last case and the time when a recurrent case is detected (Panel B). Then a further small increase (Panel C) gets the effective reproduction number continuously below one so that eradication is stable and only small outbreaks would occur if there were a new introduction.

Panels D–F show a similar pattern of the total effective reproduction number staying near one during the ramp up for intermediate waning. But given waning, the forces driving *R*_EffTot_ back up toward one now mainly arise from reinfections in individuals with waned immunity. These reinfections replace first infections in both the groups under the vaccination age and over it. The decrease in the force of infection in the vaccination age group means that vaccination levels must be higher to get the same decrease in the effective reproduction number.

A final thing to notice in the intermediate waning panels of Fig. 5 is that once eradication is achieved, further waning raises the contribution of reinfections so that it only takes very low levels of susceptibility in the under-five age group to get the effective reproduction number above one. Thus, eradication in the presence of waning is unstable.

In Fig. 6 we see how a delay affects the dynamics in the absence or presence of waning. Both in the presence and in the absence of waning, a delay causes a decrease in the size of the drop in the total effective reproduction number when vaccination is boosted. In both cases, that decrease occurs because the contribution to the effective reproduction number by the age group getting vaccinated decreases and is replaced by contributions from other groups. When there is no waning, it is the contribution from the older age group who did not get vaccinated that increases during the delay. With ongoing waning, it is primarily the contribution from older individuals whose immunity has waned that increases. Again, we see here that when there is ongoing waning, it takes much higher levels of vaccination to get elimination of polio cases and eradication of transmission.

Note that the difference in the compensating groups implies different policies for addressing a failure to eliminate polio cases or a recurrence of polio cases after their elimination. If there is no waning, then the increased susceptible individuals over age 5 will all have accumulated during the delay and thus young people should be targeted for special control programs. But in the presence of ongoing waning, it is the age groups that have passed the most time since vaccination or natural infection whose immunity is most likely to have waned. Thus, older, rather than younger, age groups should be targeted.

Fig. 7 shows how waning patterns affect the outcome of eradication efforts given nearly identical patterns of decrease in polio cases as shown in Fig. 3. All three panels have waning that produces the same average age of first infection before vaccination is begun given the same level of transmissibility as shown in Table 1. We focus on three times on the horizontal axis of each panel: (1) before vaccination, (2) during the ramp up, (3) after the boost.

Before vaccination is begun, all three waning scenarios have the same fractions of age and immunity groups that contribute to the total endemic effective reproduction number of one. During the ramp up, as the depth of waning increases it takes higher vaccination levels to get to a first infection prevalence of 300. The largest jump is between fast-shallow and intermediate waning. Likewise, there is a considerable difference between fast shallow and either intermediate or slow deep waning regarding the buildup of the fraction of the effective reproduction number that comes from reinfected individuals. Fast shallow waning levels off so that there is no ongoing waning after the first few years but any ongoing waning, whether it is a little or a lot, makes a big difference in dynamics.

Finally, we look at the response to the boost given the same final level of vaccination. The difference between those scenarios with and without ongoing waning is dramatic. There is very little rise in the contribution of reinfections for the fast-shallow scenario and consequently the effective reproduction number drops dramatically and eradication is achieved rapidly and stably. In contrast, the rise in the contribution of reinfections is higher in the intermediate and slow-deep scenarios, and in both, eradication is achieved slowly if at all, and unstably. The difference between intermediate waning and slow deep waning is enough to shift the end of SC from mathematically unstable eradication to recurrence of detected paralytic cases.

### 3.5 Patterns of SC with increasing boosts to higher final vaccination levels with and without delays

Examining Fig. 5, Fig. 6, Fig. 7 and seeing where each of the specific cases in these figures fall on the curves in Fig. 4 should now give the reader a better understanding of what Fig. 4 represents. Note in Fig. 4 that the range of vaccination levels that result in prolonged silent circulation increases with increased waning across panels A–D and E–H. It also increases in each panel between the no delay case and the delay case with a 20-year ramp up to levels of vaccination that still allow for detectable paralytic cases. Finally, it also increases with increased transmission as seen by comparing A and E, B and F, C and G, and D and H. There is little difference between no waning and fast-shallow waning scenarios but bigger differences between fast-shallow waning and intermediate level waning. Those differences are augmented given vaccination delays and greater transmission.

A big determinant of how likely we are to encounter prolonged silent circulation in the real world is the width of the vaccination levels across which there is prolonged silent circulation. If vaccination levels must be held in a narrow range for there to be prolonged silent circulation, then natural fluctuations and chance effects will eliminate that risk. Thus, one conclusion from Fig. 4 is that prolonged silent circulation will become increasingly likely in the real world as the delay in achieving effective vaccination levels increases and as the amount of ongoing waning increases. These two factors are more important than the level of transmissibility in determining silent circulation risks. The effects of these factors, however, are greater at higher levels of transmissibility.

This is important because empirical experience with prolonged silent circulation risks is based on experience in settings with lower transmission levels and shorter times between beginning vaccination efforts and ending polio cases than are characteristic of the currently or recently endemic countries. Thus, there is reason to expect that empirical experience may not be a good guide for setting the time period after the last polio case at which we are confident that there is no ongoing silent circulation.

Another thing to note is that higher vaccination rates are required to eliminate paralytic cases as the depth of waning is increased. This is noted by seeing where the curves arise above the zero level. The effects of delays in reaching needed vaccination levels and of increasing transmission on the levels of vaccination needed to eliminate detections of paralytic cases are analogous to their effects on increasing the range of vaccination levels with prolonged silent circulation.

Another observation is that, in our model, prolonged silent circulation almost always ends with a recurrent case. Once a high enough level of vaccination is reached to eradicate infection, there is only a narrow range of vaccination levels where it takes more than three years of SC before eradication levels are reached. As final vaccination levels in our model are raised, the first eventful occurrence is that more than a year passes without a paralytic case so that our curves rise above the zero level. Then it takes only a tiny further increase in vaccination levels to get the duration of SC above 3 years. With higher vaccination levels, the time of a recurrent case is pushed further into the future so the duration of SC increases. But the duration of SC is limited by finally reaching a level where eradication occurs. The peaks in Fig. 4 curves are always cases where SC ends with a recurrent case.

A final observation is that when eradication occurs, whether it is stable eradication where new introductions only cause small outbreaks, or unstable eradication where long chains of transmission can be sustained, depends strongly on the depth of waning. Eradication in the no waning and fast-shallow waning scenarios is mostly stable waning while stable waning is almost never seen with intermediate or slow-deep waning. Without ongoing waning after a few years, there is not enough of a buildup of partially susceptible individuals with waned immunity after eradication occurs to get the total effective reproduction number above one should there be a reintroduced case.

### 3.6 A different graph for the same phenomenon

**Fig. 8:**
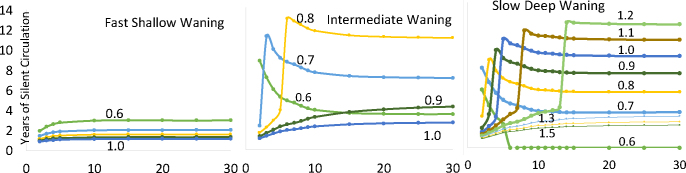
Years of silent circulation (vertical axis) by type of waning (in different panels), years between beginning a vaccination program and lowering first infection prevalence to 300 per million, (horizontal axis) and the rate per year to which vaccination of children under 5 is boosted when the prevalence of first infection reaches 300 (labels on each line). The horizontal axis is plotted only at a 2-year delay and above.

Instead of graphing the final vaccination level in a continuous manner and the delay in time as discrete, it is instructive to switch to make the delay more continuous and the final vaccination level more discrete. We do this in Fig. 8. For that figure, for each delay time we calculate the vaccination rate at the end of the delay period to give a prevalence of first infection of 300 individuals in our population of one million.

In Fig. 8, the effect of increasing waning depth on the range of vaccination levels across which there is prolonged silent circulation is seen just as it is in Fig. 4. In fast-shallow waning there is no prolonged silent circulation at a vaccination rate in children under five above an effective vaccination level of 0.6 per child per year. In intermediate waning, there is prolonged silent circulation from 0.6 to above 0.8. And in slow deep waning, there is prolonged silent circulation from 0.6 to above 1.2.

This graphic, however, shows something not seen in Fig. 4. It uncovers in more detail how the length of the delay in finally achieving effective vaccination levels interacts with both waning depth and final vaccination levels to affect the duration of SC. As delay time increases in the presence of ongoing waning beyond the first few years (intermediate and slow-deep waning in this figure), there is a sharp transition from a specific vaccination level resulting in relatively short SC that ends in eradication to much longer SC that ends in a recurrent case. As the delay time increases, both the vaccination levels that undergo this transition increase and the duration of the SC increase. At some vaccination levels after a boost, there appears to be a threshold for the length of the delay where reinfections cause prolonged low-level SC. This threshold, although generated by reinfections, is different from the reinfection threshold discussed by (Gomes et al., 2005). The threshold occurs as reinfection is increased, as in Gomes et al. But here the threshold is for SC, not any circulation.

### 3.7 Serotype differences in silent circulation potential

**Fig. 9:**
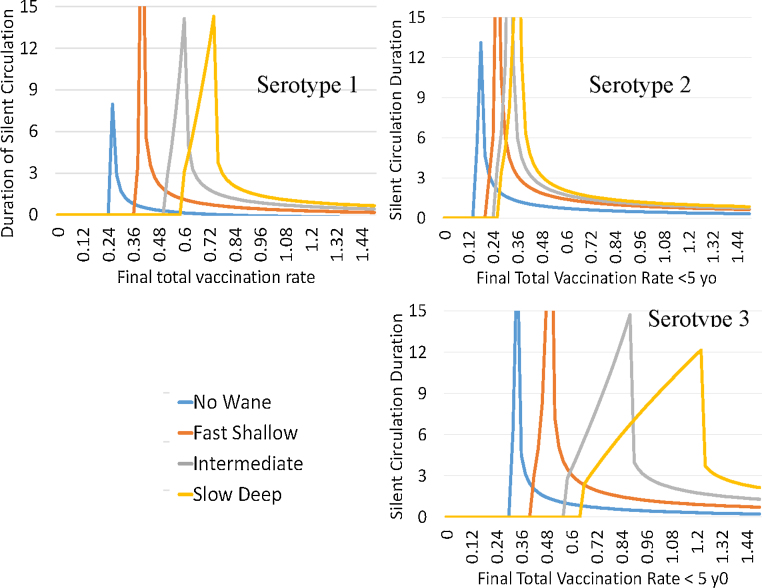
Length of silent circulation by final total vaccination rates in less than five year olds and waning scenarios for different serotypes. Settings include a 20 year ramp up time delay and high transmission.

Our model assumes that serotypes differ only in their first infections to paralytic infections ratio (IPR) and in their OPV transmissibility to WPV transmissibility ratio or vaccine transmissibility which we label as VT. We use values of VT of 0.37, 0.57, and 0.25 and values of IPR of 200, 2000, and 1000 for types 1, 2, and 3 respectively. These values are in the range used by other modelers (Duintjer Tebbens et al., 2013c). In this model, Serotype 3 is the hardest to eradicate and Serotype 2 the easiest (Fig. 9). The risks are smaller for the no waning scenario and the fast-shallow waning scenario and the changes in silent circulation for these first two waning scenarios are mostly in the level of vaccination where prolonged silent circulation is seen rather than the width of the vaccination range across which it occurs. But for intermediate and slow-deep waning, the effects on the width of the vaccination interval resulting in prolonged silent circulation is quite large.

**Fig. 10:**
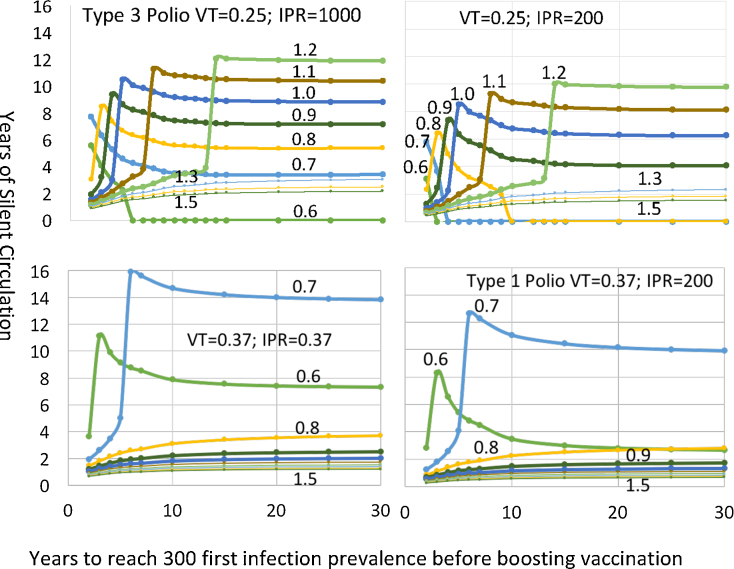
Years of silent circulation for slow deep waning by the ratio of OPV transmissibility over WPV transmissibility, infection to paralysis ratio, and the number of years to ramp up vaccination just enough to reach a prevalence of 300 first infections before boosting vaccination to the final levels indicated by the labels above the lines.

To separate the effects of the IPR and VT we compared settings starting with the serotype 3 settings of 1000 and 0.25 and changed them to 1000 and 0.37 and 200 and 0.25 so that we could see more clearly what leads to the difference between serotype 1 and serotype 3. The graphs with continuous delay times and discrete vaccination levels show very clearly the different effects of the two parameters varied. In Fig. 10 we see that the big effect on the risk of SC is the lower vaccine transmissibility (VT) rather than the higher IPR. A lower IPR lowers the duration of SC given that there will be a recurrent case because it makes it easier to detect the recurrent case. Inadequate AFPS would have the same effect as a high IPR. But a lower VT leads to long duration SC at higher vaccination rates.

This graph also shows how the two causes of SC (poor AFPS and reinfection transmission) interact. In our model, lowering VT increases reinfections because less vaccine transmission means less boosting of waned immunity. Raising the IPR could be a result of poor AFPS. In Fig. 10 we see that poor AFPS slightly lengthens any silent circulation generated by the mechanisms we have illustrated in our analysis. But increasing waning by decreasing vaccine boosting has two effects. First, for any given vaccination boost amount after a delay in eliminating cases, if the SC threshold is passed, SC duration levels out at a shorter value. Compare the 0.7 vaccination boost level to see that. That is because more waning shortens the time to a recurrent case. But the second effect could be more important. More waning means that the silent circulation threshold can be passed with much higher vaccination boosts. That is because vaccination boosts affect the completely susceptible children being vaccinated less because reinfections have displaced them in the overall effective reproduction number near one before the boost in vaccination. Consequently, as illustrated in Fig. 5, Fig. 6, Fig. 7, the effective reproduction is dropped less. It is also dropped for less time because there are more individuals with waned immunity to bring the effective reproduction number back up toward one.

### 3.8 Levels of prolonged silent circulation

Prolonged silent circulation that eventually results in eradication in the situations we examined had a maximum prevalence of wild polio virus infections summing across first and repeat infections of 110 per million or lower for the slow deep waning and 81 for the intermediate waning. Those levels were highest at the time of the last polio case and decreased progressively with time unless there was a rebound leading to a recurrence of infection before there was eradication. For first infections those top values are 50 and 32. It could thus take a considerable time to see a paralytic polio case and missing a polio case in AFPS surveillance could double that time.

For the scenarios that ended in a recurrent case, the maximum prevalence occurred when the recurrent case appeared right after three years. For both slow deep and intermediate waning, the prevalence was 200 per million at the time of the last polio case. It went down for a year, and then came back just before three years to 500.

These levels would take extensive environmental surveillance (ES) to detect. Not only would ES need to detect a single reinfection in every population of 10,000, but the detection sensitivity would have to be more than 10 times greater than that needed to detect a first infection. Thus, ES as currently being implemented is likely to miss the prolonged low level silent circulation that arises in our model.

### 3.9 Levels of vaccination in older age groups needed to avoid prolonged silent circulation.

To evaluate the effect of vaccinating older age groups before OPV use is stopped, we determined the rate at which the five and older age group would have to be vaccinated to bring about eradication in less than three years from the last polio case. The worst-case from Fig. 4 is the high transmission scenario with slow deep waning after a 20-year delay. In this situation, only 8% of the ≥5 population needs to be vaccinated per year to eradicate infection within three years of the last polio case. At higher final vaccination levels of the <5 population it takes less. If the ramp up goes to a lower prevalence of polio before the vaccination is boosted to final levels, it also takes less vaccination in the older age group to ensure that silent circulation is not prolonged.

Since the model analyzed here does not break down the ≥5 population into adults and older children, we cannot say what age groups are the most important to vaccinate to boost waned infections. But that would seem to be adults and not older children since the adults will have experienced more time since getting directly vaccinated with OPV and have less exposure to children who have been recently vaccinated.

When vaccination of the older age groups is only during the third year after the last polio case, it takes considerably more vaccination of the older age groups to eliminate prolonged silent circulation. In our worst-case scenario discussed above, it takes nearly 1.4 vaccinations per adult during that final year. But if vaccination of the older age groups occurred during the second and third years after the last polio case, only 29% of the older age group would need to be vaccinated per year.

## 4 Discussion

Our analyses develop theory about the final stage of polio eradication that involves stopping OPV use. The implications of that theory are that dynamics that have not been previously identified could generate prolonged low-level silent circulation (SC). We only examined a simple model that helps us develop the needed theory. The task of using that theory to better guide polio eradication decisions is left to the future.

### 4.1 Major findings

#### 4.1.1 The dynamics behind SC uncovered by our model analysis

In our model, waning immunity that continues for many years (ongoing waning) leads to prolonged, low-level SC under conditions that emerge only after long delays in lowering polio levels in high transmission countries. Purely short term waning does not have this effect. We adjusted the total amount of waning so that every waning scenario we examined generated the same average age of infection in the pre-vaccine era. We show that dynamics driven by forces that bring the effective reproduction number back to a value of one lie behind this risk generation. Vaccination reduces WPV infections that boost waned immunity while increasing OPV infections to a lesser degree. Consequently, older individuals not subject to vaccination make a greater contribution to the effective reproduction number while children in the ages receiving vaccination make a lower contribution. Therefore, vaccination lowers the effective reproduction number less in the presence of ongoing waning given many years of failure to eliminate polio. At the same time, vaccination raises the number of reinfections which are not detected by AFPS. These two phenomena increase the duration of SC over time.

Another way of thinking about these dynamics is to consider what is keeping the *R*_EffTot_ near one when vaccination has been ramped up slowly. When vaccination of children lowers the *R*_EffTot_ below one a force driving the effective reproduction back up toward one comes from reinfections in individuals with waned immunity. This force increases with increasing levels of vaccination since fewer individuals with waned immunity will have their immunity boosted by WPV infections. That means children under age five are replaced in *R*_EffTot_ by older individuals with reinfections. It thus takes higher rates of vaccination to get the same lowering of *R*_EffTot_. This phenomenon likely explains why such high levels of vaccination are or were required to eliminate cases in India, Nigeria, and Pakistan.

In the real world, we cannot see this effect of decreased boosting of waned immunity from increased vaccination. That is because in the absence of environmental surveillance (ES) we never detect reinfections. And even with ES, we never know if we are detecting viruses from first infections or reinfections.

Another important effect of ongoing waning combined with slow ramp up to adequate vaccination levels is that it increases the gap between vaccination rates that eliminate paralytic polio cases and those that eradicate infection. This makes it more likely that SC will persist. No matter how a model is made more realistic, this effect seems likely to persist and create conditions where the risk of prolonged low-level silent circulation could have emerged only recently in the eradication program. The remaining question then is whether the total accumulation of individuals with waned immunity is enough to sustain SC. Our model examined only homogeneous populations deterministically. In stochastic models with localized contact, spotty, local, and migratory transmission at even lower levels than found by our deterministic models is likely. In that case, local die-out of transmission will shorten the duration of SC. However, we hypothesize that the same basic dynamics we have elucidated that lead to prolonged low-level SC in our models will have similar effects in such models.

#### 4.1.2 SC from these mechanisms has low infection levels

Because vaccination that can eliminate paralytic poliomyelitis greatly lowers the contribution of first infections to the effective reproduction number, the levels of infection that sustain SC in our models are low. Such low levels might require extensive environmental surveillance (ES) to be detected. But increased acute flaccid paralysis surveillance (AFPS) is less likely to detect silent circulation.

#### 4.1.3 Effects of transmission potential on SC

In our model, even at the deep level of waning we examined, there is little potential for prolonged SC at levels of transmission that characterized industrialized countries before 1950 using the criteria of (Fine and Carneiro, 1999). Within their range of poor developing countries, however, higher transmission potential augmented the effects of vaccination delays, of reduced OPV transmission, and of waning in generating SC.

#### 4.1.4 Effects of OPV transmission and AFPS on SC

We demonstrated how infection to paralysis ratios (IPR) and the relative transmission of OPV to WPV both independently affect SC (Fig. 10). Poor AFPS effectively raises the IPR. But the effect of this on SC is modest compared to the effect of lower OPV transmission. That is because lower OPV transmission together with long times to reach adequate vaccination levels can raise the number of individuals with waned immunity to a threshold level that sustains prolonged silent circulation.

#### 4.1.5 Strategies to prevent SC

We found that if vaccination of all age groups was conducted in our model during the three years between the last cases and the declaration of eradication, SC duration could be lowered considerably by vaccinating small fractions of the over-five population. Three years were needed to ensure that the effective reproduction number was kept low enough for long enough to achieve eradication. One year required vaccination levels that might be difficult to achieve.

#### 4.1.6 The non-identifiability of SC risks using only AFPS data

A key finding in our study is that the potential for prolonged SC could be missed by models fitted only to polio case data. Fig. 3 shows that the different waning scenarios that have dramatic effects on the duration of SC (as seen in Fig. 4) can have only small differences in first infection patterns. The differences in Fig. 3 would not be detectable given expected noise from imperfect surveillance and chance events. In other words, we show that nearly identical patterns of decreasing cases on the path to polio elimination can be consistent with models having either low or high risks of prolonged SC. Thus, models fitted to AFPS data alone will have no practical capacity to predict how long SC will continue after the last polio case. This may explain why two recently published studies found little risk of prolonged silent circulation (Famulare, 2015, Kalkowska et al., 2015).

### 4.2 Relating our analyses to other studies

#### 4.2.1 Kid Risk analyses

The model in this paper is not only simpler than other models currently being used to guide polio eradication. It is also simpler than our previous published models in [Mayer]. We became convinced from our exploration of the model in (Mayer et al., 2013) that important effects on SC risks were being missed by the detailed models developed by the Kid Risk modeling group as exemplified in (Duintjer Tebbens et al., 2013c). To clarify the dynamics behind this, we formulated a model where the contributions of different parameters and population groups could be mathematically separated. To guide policy, the simplifying assumptions in our model need to be realistically relaxed to see if that affects specific policy decisions. A good starting point for identifying simplifying assumptions that are important to relax and others that are unlikely to make any difference is to understand population dynamics level mechanisms well. Our analysis pursues that goal as a first step in the process of inference robustness assessment (Koopman and Foxman, 2016, Koopman et al., 2016).

The Kid Risk models include many realistic details and exclude many others. Their assessment of what was known and unknown regarding factors affecting polio transmission dynamics (Duintjer Tebbens et al., 2013a, Duintjer Tebbens et al., 2013b) showed that little was known about waning, other than that there was definite waning after first infections by OPV and WPV in young children. Then using their informal fitting methodology (Duintjer Tebbens et al., 2013c) they found that only waning parameters that eliminated all ongoing waning seemed to fit AFPS data in their studies (Duintjer Tebbens et al., 2013c). Consequently, they have used these waning parameters in all subsequent work. This may not create problems for using their models to inform decisions about control before polio cases have been eliminated. But our analyses show that it is a problem for their studies informing final endgame plans.

The crux of the issue lies in how solid their inference is that waning does not continue beyond a short initial period. We suspect that they found they could fit their model only with parameters that eliminated ongoing waning because they used a highly complex model with many unknown or poorly specified parameters that they fitted using undocumented informal procedures. For example, they adjust contributions of oral and anal transmission to fit patterns from different localities that might have different overall levels of transmission and different contact patterns affecting infection spread. If they set these parameters and infection to paralysis ratios before they fit waning parameters, or before they fit contact pattern parameters, then only waning parameters that do not permit ongoing waning will fit AFPS data. Other similar fitting issues might also explain why they found that only parameters without ongoing waning fit their data. Unfortunately, since they do not make available either their code or the data points to which they informally find fitting parameters, as is needed for good science (Nosek et al., 2015), we could not directly assess their methods to see if our concerns were correct.

#### 4.2.2 Institute for disease modeling paper

A recent paper (Famulare et al., 2016) proposes a model of waning processes that can be informed by diverse observations including OPV transmission to direct contacts after vaccination, OPV excretion levels and durations after OPV administration given various initial immunity levels, and dose-response observations for OPV and the effects of immunity levels on dose-response. Rather than using direct antibody measures they used an “OPV-equivalent antibody titer” inferred from limited data. They then fit the inferred antibody level to time using a power law relationship between antibody level and time. While the indirect data used is weak and fits to the power law relationships are correspondingly weak, this work represents the best available data-informed look at waning immunity against polio transmission.

The model of waning immunity that emerges from this work is quite different from the model in this paper. It has continuous waning as in our older paper (Mayer et al., 2013) rather than the one-step waning in this paper. It encompasses different degrees of boosting of immunity to different levels that depend upon the starting level. Thus, cumulative immunity can be increased significantly by sequential infections. Consequently, the distribution of waned immunity by age will be greater in younger ages and less in older ages than is the case for the waning model in this paper. The power law relationships inferred between immunity levels and time lead to fast waning early and slower waning later. The decrease in waning over time is considerable. But there seems to be plenty of ongoing waning in their formulation to generate the dynamics we have illustrated that lead to prolonged low-level SC.

This waning formulation has the virtue of incorporating different aspects of waning about which it is possible to develop individual level experiments and population level studies that can directly inform model elements. That has two advantages. Fewer parameters must be fit to population levels of infection and more theory can be used when fitting remaining parameters to data. Consequently, parameter identifiability is improved. Finally, waning is conceptualized with greater mechanistic detail. This facilitates the process of inference robustness assessment (Koopman and Foxman, 2016, Koopman et al., 2016).

#### 4.2.3 Polio strains in Pakistan

Alam et al. (2016) have described polio strains in Pakistan detected by either ES or AFPS. They find that a wide variety of Type 1 polio strains are still being isolated and that different strains are geographically limited. They describe long periods where some strains are identified only in ES and not in AFPS. These are in areas with good vaccination. This provides some support to the idea that waning dynamics might be acting to increase potential for prolonged low-level SC.

Another aspect of that study, however, may indicate that some aspects of our model do not represent what is happening in Pakistan. ES has found only type 1 and not any type 3 strains. In our model, type 3 strains had more potential for prolonged low-level SC than did type one. That inconsistency might be explained in different ways. First, it might be that WPV3 has considerably lower transmissibility than type 1. That in turn would diminish the effect of the lower transmissibility of OPV3 compared to OPV1. (Duintjer Tebbens et al., 2013a, Duintjer Tebbens et al., 2013b, Duintjer Tebbens et al., 2013c) formulated only slight differences between WPV3 and WPV1. But the data for that formulation are weak. Second, it might be that the silent circulation of WPV3 is very low-level, consistent with our model results. In that case the sensitivity of ES, which is quite low, may not be sufficient to detect prolonged low-level SC. Third, it might be that the dynamics of boosting as formulated in Famulare et al. (2016) are greater for WPV3 than WPV1. Or the dynamics of waning might be higher.

#### 4.2.4 Other studies indicating the potential for prolonged low-level SC

Other studies indicate ongoing waning occurs in the real world. A study of elderly Dutch found that they had durations and levels of OPV excretion after vaccination that were quite long and high (Abbink et al., 2005). A study in India of excretion after OPV administration showed a loss of immunity between ages 5 and 10 (Jafari et al., 2014) that could be explained by ongoing immunity.

The potential for SC is illustrated by “orphan” viruses that do not show close genetic links to other recently identified viruses (Hagan et al., 2015, Porter et al., 2015). Viruses might be detected by AFPS or environmental surveillance (ES). Orphan viruses might arise because paralytic polio cases were missed due to inadequate AFPS and/or because reinfection transmissions, that are unlikely to cause paralysis, sustained circulation. Both factors might be acting in many situations. The dynamics we illustrate in this article show a potential for increasing this latter cause over time in high transmission areas. The most dramatic detection of an orphan virus was the discovery in 2016 of polio cases in Nigeria after more than a year without any detected case in the country. The orphan viruses in this case had not been detected for more than four years (Roberts, 2016). In this case, ES in Northeastern Nigeria detected a cVDPV2 in Nigeria after all OPV2 use had been stopped globally (Etsano et al., 2016). That stimulated an intensive effort to vaccinate in an area where Boko Haram had severely hampered vaccination and surveillance activities. This effort uncovered the polio cases.

ES detected an SC epidemic of WPV1 in Israel in 2013 (Tulchinsky et al., 2013). This SC epidemic occurred because Israel switched to using only IPV in 2005. IPV without prior OPV cannot stop WPV transmission. The resulting SC epidemic was different from the low-level SC we have modeled after long use of OPV. The IPV use caused accumulation of large numbers of children susceptible to infection but not paralysis. Consequently, there were very high levels of infection in Bedouin children in affected villages (Manor et al., 2014, Shulman et al., 2014a, Shulman et al., 2014b, Shulman et al., 2014c). Within Israel, administering OPV only to children who had received only IPV successfully eliminated virus transmission without a single paralytic case. The virus came to Israel from Pakistan. It had been previously detected by ES in Egypt but caused no polio cases there. The transmissions carrying virus across these long distances seem more likely to involve older individuals experiencing reinfections. After the Israeli silent epidemic the same virus strain caused polio cases in Syria and Iraq (Arie, 2014, Aylward and Alwan, 2014). While the main SC epidemic in Israel does not derive from mechanisms generating SC in the model examined in this paper, the long path from Pakistan and eventually to Iraq and Syria could be related to those mechanisms.

### 4.3 Using ES to guide polio eradication end-stage decisions

The dynamics we have elucidated indicate that a strict three year criteria for declaring complete eradication could be inadequate. The origin of the 3 year criterion is largely empirically based on experience in Latin America (Debanne and Rowland, 1998). Its initial theoretical support used models that assumed acquired immunity never wanes (Eichner and Dietz, 1996, Kalkowska et al., 2012). Recent model analyses assumed that waning only occurs in the first few years after infection (Famulare et al., 2016, Kalkowska et al., 2015). We believe that conditions leading to SC deserve to be studied in a way that allows for a more informed assessment of the chances that SC exists than an arbitrary three years since the last detected polio case.

We propose that ES be analyzed in a new way to assess SC risks. The current state of ES and the plans for expanding it are described in (GPEI, 2015). ES has already been used to discover long local SC of polio in Pakistan (Alam et al., 2016). In Nigeria ES has demonstrated its capacity to guide public health actions (Johnson Muluh et al., 2016). It detected a cVDPV2 that stimulated a special vaccination program after OPV2 use had been ceased in the rest of the world (Etsano et al., 2016). That in turn led to the discovery of new polio cases (Roberts, 2016). But ES is still not widely used. It covers relatively small populations and therefore would likely miss the low levels of infection generated in our models. Assessing the risks of silent circulation at the time of OPV cessation, therefore, cannot only depend on ES samples. The current use of ES data is just to indicate whether silent circulation exists and not to estimate the chances there is SC.

We propose that analyzing combined ES and AFPS data using transmission models with waning immunity parameters would improve assessment of SC risks. We hypothesize that parameters not identifiable from AFPS or ES data alone can become identifiable when using ES and AFPS data jointly. The reason for this increased identifiability is that AFPS and ES data have different identifiability tradeoffs between parameter values. Given only AFPS data, the effects of parameters that increase waning can be counteracted by increasing both transmission parameters and first infection to paralysis ratios. Given ES data, that is not the case because transmission parameter increases will be directly reflected by ES data. Thus, ES data can pin down parameters that can vary widely when fitting just AFPS data. ES data alone, however, is too sparse to provide evidence against the low levels of polio virus circulation sustained by reinfection seen in our models.

The complementarity of ES and AFPS data should help assess the potential for SC in locations with different conditions such as: history of AFPS polio detections, AFPS adequacy (GPEI, 2017), water-sanitation-hygiene (WaSH) conditions (Eisenberg et al., 2012), and the history of vaccination levels. That will help locate ES where it will be most informative. Currently sites are being located where the most problems in eliminating polio cases have been experienced (Johnson Muluh et al., 2016). But one implication of older age groups sustaining prolonged low-level SC is that populations not exposed to OPV or WPV from kids might have more waned immunity and thus be more likely to sustain reinfection transmission chains given high transmission conditions.

Statistical analyses should be designed to reduce the uncertainty in values of polio model parameters that most affect SC. Inference methods for partially observed stochastic dynamic systems (King et al., 2016) are appropriate for this. They will provide a statistical basis for assessing the probability of SC and thus better inform when OPV should be stopped. The models used should realistically relax the extreme simplifying assumptions of the models used in this paper. This should be done in an inference robustness assessment framework (Koopman et al., 2016). Sequence information could be used to make more precise estimation of the parameters affecting SC by analyzing each strain separately in a single analysis using newly developed methods (Koopman and Foxman, 2016).

This type of statistical analysis becomes increasingly important over time because as the delay time between starting vaccination and eliminating polio cases increases, ever higher vaccination levels can generate ever longer SC if there is not a large sudden increase in vaccination levels. It is not hard to envision a process in which countries with long delays step up their vaccination level to eliminate paralytic cases. As they take more time to do so, the duration of SC increases. If countries can get vaccination coverage and rates above the thresholds that lead to eradication, the global eradication effort will be successful. But because the duration of silent circulation increases with increasing delay time, it will be increasingly difficult to distinguish whether the lack of paralytic cases means eradication is on track or the future may involve a recurrence of polio.
