# Supplementary material for: Dynamics affecting the risk of silent circulation when oral polio vaccination is stopped
Source: Epidemics. 2017 Sep;20:21–36. doi: 10.1016/j.epidem.2017.02.013 (PMC5608688; doi:10.1016/j.epidem.2017.02.013)
Supplement: Supplementary file 1 [file mmc1.pdf]

1 Model, with code excerpts

This presentation is intended to make the code more understandable while revealing the more clearly the mathematical structure of the equations. To this end it uses variable and parameter names in the code rather than presenting the equations in standard mathematical form. It eliminates several complex details in the code that give it a capacity to examine more than was examined in the paper. For example, the code (with very minor changes) can be used to examine what happens when OPV vaccination is stopped, how different definitions for key events like the time of the last polio case, the time of eradication, or the time of recurrence affect the inferences made, how different waning effects on susceptibility, infection duration, and contagiousness can affect the inferences made, and how long it takes to get a detectable case if all OPV use is stopped while there is still prolonged low level silent circulation. A more standard mathematical formulation of the model is presented in the Section 2 of this appendix.

1.1 Model Variables

Initial values are not important for the analyses presented in the paper because we run the system to equilibrium before vaccination begins.
S[1..n+1] : Susceptible population in the child age group (first n indices) and in the adult age group (n+1)
WPV1[1..n+1] : WPV infected for the first time by age group (If in Figure 1)
WPVR[1..n+1] : Repeated WPV infection by age group (Ir in Figure 1)
OPV1[1..n+1] : OPV infected for the first time by age group (Vf in Figure 1)
OPVR[1..n+1] : Repeated OPV infection by age group (Vr in Figure 1)
R1[1..n+1] : First stage recovered compartment with no susceptibility to infection (R in Figure 1)
R2[1..n+1] : Second stage recovered compartment after waning (P in Figure 1)

1.2 Directly set modelParameters

Values are those used in the paper, but analyses performed often included broader parameters.
n = 40 : Number of compartments in first five years of age
m = 0.02 : Birth and death rates per year in both children and older
VaccRt1 = [.01-2] Rate of effective OPV vaccination in <5 year olds at the end of the initial ramp up.
VaccIncTm = [0-20] : The duration of the vaccination ramp up.
VaccRt2 = [0-5] : The amount of raise in vaccination rate at the end of the vaccine ramp up.
VEnd = [3-200] :Number of years after polio case elimination that all vaccination is stopped.
VAdltBgn = [0-3] : Number of years after polio case elimination that adult vaccination begins.
VacRtAdlt = [0-1.5] : Rate of effective OPV vaccination in adults. This runs from VAdltBgn to Vend.
PopSize = 10ˆ6 : The total size of the population.
ErLv = 1 : The number of prevalent WPV infections at which eradication is achieved and the force of WPV infection is set to zero.
InfPerAFP = [200-2000] : The number of first infections per paralytic polio case
gW1 = 13 : Rate of recovery from first WPV infections.

RelR0OPVoWPV = [0.25-0.57] : Relative transmission potential of OPV / WPV (type 1=0.37, type 2 = 0.57, Type 3 = 0.25).
κ = [0.01-0.9] : We set three different parameters equal to this for the paper. These are the fraction of susceptibility in people with waned immunity compared to people with no immunity (SucRt1ratio), the fraction of contagiousness in people with waned immunity who get reinfected compared to those with no immunity (RelContRt1), and the fraction of the average duration of infection in those who had waned immunity when infected compared to those who had no immunity (1 / Rec1tRratio).
tp = .5 : transmission probability given a contact
c = [30-300] : Contacts per individual per year. This is set in the BM code by setting R0CBd and tp.
WnRt = [0.02-0.2] : The rate of waning from R1 to R2 (R to P in Figure 1) compartments

1.3 Derived parameters

$$\text{LmbdaW} = \left(\sum_i \text{WPV1}_i + \sum_i \text{WPVR}_i * \text{RelContRt1} \right) /\text{TotPop}$$

$$\text{LmbdaO} = \left(\sum_i \text{OPV1}_i * \text{RelContOtW} + \sum_i \text{OPVR}_i * \text{RelContOtW} * \text{RelContRt1} \right) /\text{TotPop}$$

These are the parts of the forces of infection from contacts with either wild type (LmbdaW) or vaccine (LmbdaO) infections.

The RelContRt1 is the relative contagiousness of repeat infections compared to first infections and is always set to κ in this paper.

The RelContOtW is the relative contagiousness of an OPV infection compared to a WPV infection and is the cube root of RelR0OPVoWPV in this paper since we set contagiousness, duration and susceptibility with the same parameter value.
gWR = gW1/ κ : The rate of recovery from repeat WPV infections.
gO1 = gW1/ RelR0OPVoWPV$^{-1/3}$: The rate of recovery from first OPV infections
gOR = gW1/ (RelR0OPVoWPV$^{-1/3}$ * κ): The rate of recovery from repeat OPV infections

VaccRtbe is the variable that describes the vaccination level for children under age 5 from the start of vaccination throughout the ramp up and continuing past the jump along the final vaccination level. The code for this is complicated because that code is designed to handle stopping the use of vaccine even though the results of such stopping were not presented in the paper.

VacRAdlt is the vaccination rate of adults during specified periods before all OPV use is stopped at designated times after the last polio case.

1.4 Model differential equations

"i" on the right side of equations refers to the age group category level in the square brackets on the left. It goes from 1 to n+1 for all n age groups under 5 and the one age group covering all older ages. The first major compartment is divided into 3 age groupings to help the reader see the relationships across the age groups more clearly. These groups are then collapsed across all age groups using "if-then-else" statements for subsequent major compartments.

d/dt (S[1]) = m*PopSize Births
- S[1]*c*tp*(LmbdaW +LmbdaO) New OPV andWPV transmissions
- S[1] * ((n / 5) +m +VaccRtbe) Age out of group, deaths, vaccinations

d/dt (S[2..n]) = S[i-1] * (n / 5) Age into group
- S[i]*c*tp*(LmbdaW +LmbdaO) New OPV andWPV transmissions
- S[i] * ((n / 5) +m +VaccRtbe) Age out of group, deaths, vaccinations

d/dt (S[n+1]) = S[n] * (n / 5) Age into group
- S[n+1]*c*tp*(LmbdaW +LmbdaO) New OPV andWPV transmissions
- S[n+1] * (m + VacRAdlt) Deaths, vaccinations

d/dt (WPV1[1..n+1]) = (if i >1 then WPV1[i-1]*n/5 else 0) Age into group
+ S[i]*c*tp*LmbdaW New WPV transmissions
- WPV1[i]*((m + gW1) Deaths and recoveries
+ (if i <n+1 then n/5 else 0)) Age out of group

d/dt (OPV1[1..n+1]) = (if i >1 then OPV1[i-1]*n/5 else 0) Age into group
+ (If i <n+1 then S[i] * VaccRtbe else 0) New vaccination take
+ S[i]*c*tp*LmbdaO Vaccine transmission
- OPV1[i]*(m + (if i <n+1 then n/5 else 0) + gO1) Death, aging, recovery
+ (if i=n+1 then S[n+1]*VacRAdlt else 0) Age out of group

d/dt (R1[1..n+1]) = (if i >1 then R1[i-1]*n/5 else 0) Age into group
+ WPV1[i] * gW1 recovery from first WPV infection
+ WPVR[i] * gWR recovery from WPV reinfection
+OPV1[i] * gO1 recovery from first OPV infection
+ OPVR[i] * gOR recovery from WPV reinfection
- R1[i] * WnRt waning out of immunity
- R1[i] * ((if i <n+1 then n/5 else 0) + m) aging and death

d/dt (R2[1..n+1]) = (if i >1 then R2[i-1]*n/5 else 0) aging into group
+ R1[i]*WnRt waning into partial susceptibility
- R2[i]*c*SucRt1ratio*tp*(LmbdaW + LmbdaO) new WPV and OPV transmissions
- R2[i] * ((if i <n+1 then (n/5) else 0) aging out of group
+(if i <n+1 then VaccRtbe*SucRt1ratio else 0) new vaccination take in children <5 yo
+ m) death
- (if i >n then R2[n+1]*VacRAdlt else 0) new vaccination take in all =>5 yo

d/dt (WPVR[1..n+1]) = (if i >1 then WPVR[i-1]*n/5 else 0) aging into group
+ R2[i]*c*tp*SucRt1ratio*LmbdaW new WPV transmission
- WPVR[i]*(m death
+ (if i <n+1 then n/5 else 0) aging out of group
+ gWR) recovery from infection

d/dt (OPVR[1..n+1]) = (if i >1 then OPVR[i-1]*n/5 else 0) aging into group

+ R2[i]*(c*tp*SucRt1ratio*LmbdaO new OPV transmission
+ (if i <n+1 then VaccRtbe*SucRt1ratio else 0)) new vaccination take in children <5 yo
- OPVR[i]*(m death
+ (if i <n+1 then n/5 else 0) aging out of group
+ gOR) recovery from infection
+ (if i >n then R2[n+1]*VacRAdlt else 0) new vaccination take in all =>5 yo

1.5 Key timing variables in the code

CaseElimTm is the time when the cumulative number of first WPV infections over the past year goes below the InfperAFP parameter value. We make this the time of the last diagnosed polio case and start counting the duration of silent circulation from that time. CaseElimTm is a variable in the code that equals time until a counter variable CumFrstInf goes below InfperAFP. Thereafter it stays fixed. CumFrstInf is a counter of first WPV infections over a moving window covering the past year.

ScndAFPTm is the time when the cumulative number of first WPV infections since CaseElimTm exceeds the InfperAFP. We make this the time of recurrence of paralytic polio cases after initial elimination of these cases. ScndAFPTm is a variable in the code that equals time until a counter variable CumFrst3Slnt exceeds InfperAFP. Thereafter it stays fixed.

Erad3Tm is the time when the overall prevalence of infection including both first infections and reinfections (TotErad3) goes below ErLv. Different definitions for this time used different variables going below ErLv. TotErad4 is a summation of first and reinfections weighted by their transmission potential. TotErad5 uses an effective size weighting.

2 The effective reproduction number

2.1 Tables of parameters and variables

Note: For the sake of clarity, and because they are not relevant to this section, we omit parameters and variables relating to case detection, silent circulation, and further interventions following the last observed polio case.

2.1.1 Parameters

Symbol	Name in code	Value	Meaning
n	n	40	Number of age categories for children under 5
μ	m	0.02	(Age-independent) Death rate
N	PopSize	1e6	Total size of the modeled population
γ	gW1	13/yr	Recovery rate for a first infection with WPV
β	c*tp	[varies]	Contagiousness for a first infection with WPV
ω	WnRt	[varies]	Waning rate
θ	RelR0OPVoWPV	[varies]	Ratio of R_0 for OPV to R_0 for WPV
T_{ramp}	VaccIncTm	[varies]	Duration of vaccination ramp-up
ρ_1	VaccRt1	[varies]	Effective rate of OPV vaccination in children under 5 at the end of vaccination ramp-up, before the boost
ρ_2	VaccRt2	[varies]	Size of the boost in effective rate of OPV vaccination of children under 5 that occurs at the end of vaccination ramp-up
κ	SucRt1ratio	[varies]	[see below]

In our model, the depth of immune waning following a live virus infection is controlled by a single parameter κ, which denotes the following three values, which we assume for the sake of simplicity to be equal: The relative susceptibility to (re)infection of individuals with waned immunity, compared to fully susceptibles; the relative duration (for individuals who recover before they die) of reinfections, compared to first infections; and the relative contagiousness (i.e. shedding rate) of reinfections, compared to first infections. Consequently, the effective reproduction number of WPV in a population consisting entirely of individuals with waned immunity would be approximately κ^3 times the basic reproduction number (i.e. the effective reproduction number in a population consisting entirely of fully susceptible individuals). We therefore treat κ^3 as the definition of the waning depth.

2.1.2 Subpopulations

Symbol	Name in code	Meaning
S_i	S[i]	Fully susceptible individuals in age category i
P_i	R2[i]	Partially susceptible (i.e. waned) individuals in age category i
R_i	R1[i]	Fully immune (i.e. unwaned) individuals in age category i
$W_{1,i}$	WPV1[i]	Individuals in age category i who are experiencing their first live virus infection, with WPV
$W_{R,i}$	WPVR[i]	Individuals in age category i who are experiencing a second or subsequent live virus infection, with WPV
$O_{1,i}$	OPV1[i]	Individuals in age category i who are experiencing their first live virus infection, with OPV
$O_{R,i}$	OPVR[i]	Individuals in age category i who are experiencing a second or subsequent live virus infection, with OPV

2.1.3 Other variables and time-varying parameters

Symbol	Name in code	Value	Meaning
λ_W	LmbdaW	$\left(\frac{1}{N}\right)\sum_{i=1}^{n+1}(W_{1,i}+\kappa W_{R,i})$	Fraction of the population that is WPV-infected, scaled by fraction of first-WPV-infection contagiousness
λ_O	LmbdaO	$\left(\frac{1}{N}\right)\sum_{i=1}^{n+1}(\theta O_{1,i}+\kappa\theta O_{R,i})$	Fraction of the population that is OPV-infected, scaled by fraction of first-WPV-infection contagiousness
ρ	VaccRtbe	$\begin{cases}0, & \text{if } t \le 0\\ \left(\frac{t}{T_{ramp}}\right)\rho_1, & \text{if } 0 \le t \le T_{ramp}\\ \rho_1+\rho_2, & \text{if } T_{ramp} < t\end{cases}$	Vaccination rate for children under five

2.2 Model equations

$$\frac{d}{dt}\left(S_i\right) = -\left(\beta\left(\lambda_W+\lambda_O\right)+\mu\right)S_i + \begin{cases} \mu N - \left(\frac{n}{5}+\rho\right)S_i, & \text{if } i=1 \\ \left(\frac{n}{5}\right)S_{i-1}, & \text{if } i=n+1 \\ \left(\frac{n}{5}\right)S_{i-1} - \left(\frac{n}{5}+\rho\right)S_i, & \text{otherwise} \end{cases}$$

$$\frac{d}{dt}\left(P_i\right) = -\left(\kappa\beta\left(\lambda_W+\lambda_O\right)+\mu\right)P_i + \omega R_i + \begin{cases} -\left(\frac{n}{5}+\kappa\rho\right)P_i, & \text{if } i=1 \\ \left(\frac{n}{5}\right)P_{i-1}, & \text{if } i=n+1 \\ \left(\frac{n}{5}\right)P_{i-1} - \left(\frac{n}{5}+\kappa\rho\right)P_i, & \text{otherwise} \end{cases}$$

$$\frac{d}{dt}\left(R_i\right) = -\left(\omega+\mu\right)R_i + \gamma W_{1,i} + \left(\frac{\gamma}{\kappa}\right)W_{R,i} + \left(\frac{\gamma}{\theta}\right)O_{1,i} + \left(\frac{\gamma}{\kappa\theta}\right)O_{R,i} + \begin{cases} -\left(\frac{n}{5}\right)R_i, & \text{if } i=1 \\ \left(\frac{n}{5}\right)R_{i-1}, & \text{if } i=n+1 \\ \left(\frac{n}{5}\right)R_{i-1} - \left(\frac{n}{5}\right)R_i, & \text{otherwise} \end{cases}$$

$$\frac{d}{dt}\left(W_{1,i}\right) = -\left(\gamma+\mu\right)W_{1,i} + \beta\lambda_W S_i + \begin{cases} -\left(\frac{n}{5}\right)W_{1,i}, & \text{if } i=1 \\ \left(\frac{n}{5}\right)W_{1,i-1}, & \text{if } i=n+1 \\ \left(\frac{n}{5}\right)W_{1,i-1} - \left(\frac{n}{5}\right)W_{1,i}, & \text{otherwise} \end{cases}$$

$$\frac{d}{dt}\left(W_{R,i}\right) = -\left(\frac{\gamma}{\kappa}+\mu\right)W_{R,i} + \kappa\beta\lambda_W P_i + \begin{cases} -\left(\frac{n}{5}\right)W_{R,i}, & \text{if } i=1 \\ \left(\frac{n}{5}\right)W_{R,i-1}, & \text{if } i=n+1 \\ \left(\frac{n}{5}\right)W_{R,i-1} - \left(\frac{n}{5}\right)W_{R,i}, & \text{otherwise} \end{cases}$$

$$\frac{d}{dt}\left(O_{1,i}\right) = -\left(\frac{\gamma}{\theta}+\mu\right)O_{1,i} + \beta\lambda_O S_i + \begin{cases} \rho S_i - \left(\frac{n}{5}\right)O_{1,i}, & \text{if } i=1 \\ \left(\frac{n}{5}\right)O_{1,i-1}, & \text{if } i=n+1 \\ \rho S_i + \left(\frac{n}{5}\right)O_{1,i-1} - \left(\frac{n}{5}\right)O_{1,i}, & \text{otherwise} \end{cases}$$

$$\frac{d}{dt}\left(O_{R,i}\right) = -\left(\frac{\gamma}{\kappa\theta}+\mu\right)O_{R,i} + \kappa\beta\lambda_O P_i + \begin{cases} -\left(\frac{n}{5}\right)O_{R,i}, & \text{if } i=1 \\ \left(\frac{n}{5}\right)O_{R,i-1}, & \text{if } i=n+1 \\ \left(\frac{n}{5}\right)O_{R,i-1} - \left(\frac{n}{5}\right)O_{R,i}, & \text{otherwise} \end{cases}$$

2.3 Approach to defining an effective reproduction number

We define a next-generation matrix effective reproduction number ($R_{\text{eff.}}$) at time t in a fashion analogous to the definition of a next-generation matrix basic reproduction number (R_0) given by Diekmann et al. (2010): $R_{\text{eff.}}$ is the dominant eigenvalue of the next-generation matrix K, whose entries $\{K_{i,j}\}$ represent the expected number of secondary infections into infected subpopulation i that an individual who is infected into infected subpopulation j (at time t) would be expected

to produce over the course of their infection, if the size of all subpopulations were somehow fixed for the duration of that infection. This last constraint is also present in the definition of an next-generation matrix R_0, being implied by the requirement that the population be at a disease-free equilibrium, apart from an infinitesimal fraction that is infected. Defined in this way, $R_{\text{eff.}} = 1$ if the system is at an *endemic* equilibrium.

2.4 Derivation of the equation for the effective reproduction number

In the equations above, none of the dynamics relevant to transmission of WPV depend directly on an individual's age category. Therefore, we can define a smaller set of susceptible and WPV-infected subpopulations by collapsing together subpopulations that only differ with respect to their ages. For understanding the dynamics of this particular sytem, however, it is useful to maintain a distinction between individuals under 5 and individuals who are 5 and older, rather than ignoring *all* distinctions of age:

$$
\begin{aligned}
S_1^* &= \sum_{i=1}^{n} S_i \\
S_2^* &= S_{n+1} \\
S_3^* &= \sum_{i=1}^{n} P_i \\
S_4^* &= P_{n+1} \\
W_1^* &= \sum_{i=1}^{n} W_{1,i} \\
W_2^* &= W_{1,n+1} \\
W_3^* &= \sum_{i=1}^{n} W_{R,i} \\
W_4^* &= W_{R,n+1}
\end{aligned}
$$

In this formulation, infections into the the i-th WPV-infected subpopulation (W_i^*) are precisely infections from the i-th susceptible subpopulation (S_i^*). When this is the case, $K_{i,j}$ is simply the product of the expected number of contacts that an individual infected into W_j^* will make with individuals in S_i^* over the course of their infection and the average probability of transmission per contact across those contacts.

Our model has several additional simplifying features as well:

- The contact rate is the same for all individuals.
- Mixing is proportional.
- The contagiousness of an individual infected into W_i^* does not vary over the course of their infection.
- The relative susceptibility of individuals in each susceptible subpopulation S_i^* does not depend on which infected subpopulation W_j^* their potential infector is in.

When all of these are true, the expression for the entries of the next-generation matrix is quite simple:

$$K_{i,j} = \beta_j D_j \sigma_i S_i^*$$

where β_j is the contagiousness of individuals in W_j^*, D_j is the average duration of an infection in W_j^*, and σ_i is the relative susceptibility to infection of individuals in S_i^*. (The values of these parameters are given in a table in the next section, although the following results do not depend on them.)

This implies that all the rows of the matrix K are multiples of each other (specifically, the i-th row is $\frac{\sigma_i S_i^*}{\sigma_j S_j^*}$ times the j-th). Thus, the rank of the matrix K (the maximum number of linearly independent rows of K) is 1. The number of non-zero eigenvalues (counting with multiplicity) of a matrix is equal to its rank; therefore K has only one non-zero eigenvalue. Consequently, the dominant eigenvalue of K is equal to the sum of the eigenvalues of K. The sum of the eigenvalues of a matrix is its trace (the sum of entries on the main diagonal). Therefore:

$$\begin{aligned} R_{\text{eff.}} &= \sum_{i=1}^{4} K_{i,i} \\ &= \sum_{i=1}^{4} \beta_i D_i \sigma_i S_i^* \end{aligned}$$

$K_{i,i}$ is simply the expected number (under the given assumptions) of secondary transmissions into W_i^* from an individual infected into that same subpopulation. Consequently, we describe it as the contribution to the effective reproduction number of that subpopulation, and obtain the equation given in the main text:

$$R_{\text{eff.}} = R_{\text{eff.First},<5} + R_{\text{eff.Subsequent},<5} + R_{\text{eff.First},\geq 5} + R_{\text{eff.Subsequent},\geq 5}$$

2.5 Derived variables used to calculate the effective reproduction number

Symbol	Value	Meaning
β_j	$\begin{cases} \beta, & j = 1,2 \\ \kappa\beta, & j = 3,4 \end{cases}$	Contagiousness of an individual in W_j^*
D_j	$\begin{cases} \frac{1}{\gamma + \mu}, & j = 1,2 \\ \frac{\kappa}{\gamma + \kappa\mu}, & j = 3,4 \end{cases}$	Mean duration of infection of an individual in W_j^*
σ_i	$\begin{cases} 1, & i = 1,2 \\ \kappa, & i = 3,4 \end{cases}$	Relative susceptibility of an individual in S_i^*

2.6 Contributions to the effective reproduction number

Symbol	Value	Meaning
$R_{\text{eff.First},<5}$	$\left(\frac{\beta}{\gamma+\mu}\right)\sum_{i=1}^{n} S_i$	Contribution of first infections in the under-five age group
$R_{\text{eff.Subsequent},<5}$	$\kappa^3\left(\frac{\beta}{\gamma+\kappa\mu}\right)\sum_{i=1}^{n} P_i$	Contribution of reinfections in the under-five age group
$R_{\text{eff.First},\geq 5}$	$\left(\frac{\beta}{\gamma+\mu}\right) S_{n+1}$	Contribution of first infections in the five-and-older age group
$R_{\text{eff.Subsequent},\geq 5}$	$\kappa^3\left(\frac{\beta}{\gamma+\kappa\mu}\right) P_{n+1}$	Contribution of reinfections in the five-and-older age group

3 The effect of waning parameters on the relative contribution of reinfections to the pre-vaccination equilibrium force of infection

3.1 Motivation

In the main text, we present four different scenarios for the high-transmission setting: one without waning of immunity following live virus infection, and three with waning of immunity, but varying in the speed and depth of that waning. (We also do this for the low transmission setting, but as discussed in Table 1 of the main text, it was the high-transmission setting that we used to fit our waning parameters for each of the three scenarios with waning; consequently, we focus exclusively on that setting in this appendix section.) All four scenarios have the same average age of first infection (A), and all three scenarios with waning have the same basic reproduction number (R_0) as well.

In this appendix section, we use those two constraints as a starting point in order to understand how the various waning parameters influence the relative contribution of first infections and reinfections to the force of infection at the endemic equilibrium. In this way, we will build a deeper understanding of the interaction between waning depth and waning rate in a broader sense.

3.2 Tables of symbols

3.2.1 Parameters

Note: For the sake of clarity, and because the focus of this section is on transmission dynamics at the pre-vaccination endemic equilibrium, the following table omits parameters related to vaccination, transmission of vaccine virus, case detection, and real or apparent elimination of transmission.

Symbol	Value	Meaning
N	1e6	Size of the modeled population
μ	0.02/yr	(Age-independent) Death rate
L	$\frac{1}{\mu}$	Average lifespan
γ	13/yr	Recovery rate for a first infection
A	[varies]	Average age at (first) infection
β	[varies]	Contagiousness for a first infection
ω	[varies]	Waning rate
λ	$\frac{1}{A} - \mu$	Force of infection operating on fully susceptible individuals, at the pre-vaccination endemic equilibrium
κ_s	[varies]	Relative susceptibility to (re)infection of individuals with waned immunity, compared to fully susceptibles
κ_d	[varies]	Relative duration (for individuals who recover before they die) of reinfections, compared to first infections
κ_c	[varies]	Relative contagiousness (shedding rate) of reinfections, compared to first infections
κ	[varies]	[see below]

In our model as actually implemented, the depth of immune waning following a live virus infection is controlled by a single parameter κ, which denotes the following three values pertaining to individuals with waned immunity, which we assume for the sake of simplicity to be equal: Relative susceptibility to reinfection (here denoted κ_s), the relative duration of reinfections (κ_d), and the relative contagiousness of reinfections (κ_c). However, in order to better show how each of these components of immune waning affect the relative contribution of reinfections to the force of infection at the pre-vaccination endemic equilibrium, we will distinguish them here. At the conclusion of this section of the appendix, we will show how the results we obtain can be simplified when all three components of immune waning are in fact equal, as is the case for all results presented in the main text.

3.2.2 States of immunity and infection

The following symbols correspond to those used for subpopulations in section 2 of this appendix, omitting the age subscripts.

Symbol	Meaning
S	Fully susceptible
W_1	Currently first-infected with WPV
W_R	Currently reinfected with WPV
R	Recovered from infection and fully immune
P	Partially susceptible, following waning of immunity

3.2.3 Probabilities and expectations

Several of the following values are derived in subsequent sections; they are presented here along with the values that follow immediately from the parameter definitions above for ease of reference.

Symbol	Value	Meaning
$\mathbf{P}(X \to Y)$	[see following entries]	Probability for an individual in state X to transition to state Y before dying
$\mathbf{P}(S \to W_1)$	$\frac{\lambda}{\lambda + \mu}$	
$\mathbf{P}(W_1 \to R)$	$\frac{\gamma}{\gamma + \mu}$	
$\mathbf{P}(R \to P)$	$\frac{\omega}{\omega + \mu}$	
$\mathbf{P}(P \to W_R)$	$\frac{\kappa_s \lambda}{\kappa_s \lambda + \mu}$	
$\mathbf{P}(S \to W_R)$	$\frac{\kappa_s \lambda^2 \gamma \omega}{(\lambda + \mu)(\gamma + \mu)(\omega + \mu)(\kappa_s \lambda + \mu)}$	
$\mathbf{P}(W_R \to W_R)$	$\frac{\kappa_s \lambda \gamma \omega}{(\gamma + \kappa_d \mu)(\omega + \mu)(\kappa_s \lambda + \mu)}$	Probability for a currently reinfected individual to recover and subsequently be reinfected again, before dying
$\mathbf{E}(W_R)$	$\frac{(\gamma + \kappa_d \mu)(\omega + \mu)(\kappa_s \lambda + \mu)}{(\gamma + \kappa_d \mu)(\omega + \mu)(\kappa_s \lambda + \mu) - \kappa_s \lambda \gamma \omega}$	Expected number of reinfections, conditional on being reinfected at least once
$\mathbf{D}(W_1)$	$\frac{1}{\gamma + \mu}$	Average duration of a first infection
$\mathbf{D}(W_R)$	$\frac{1}{\kappa_d^{-1} \gamma + \mu}$	Average duration of a reinfection

3.3 Basic Approach

Our model has an exponential population structure, vulnerability to infection that does not depend on age, and homoegenous mixing. Consequently, at the endemic equilibrium, the following relationship between the average age of infection, the death rate, and the force of infection holds (Dietz 1993):

$$A = \frac{1}{\lambda + \mu}$$

Because we fix both the average age of infection and the death rate, this means that the force of infection can be treated as a derived parameter:

$$\lambda = \frac{1}{A} - \mu$$

In order to focus on the effects of the waning parameters, we will begin by treating the (first-infection) contagiousness parameter β as fixed. As we will see in subsequent sections, that parameter will not appear in the equation for the relative contribution of reinfections to the endemic force of infection. This does not mean that it is irrelevant, but rather that it is a free parameter that can be set (as it in fact is, in our model) to the value that produces the correct equilibrium force of infection.

3.4 Derivation

3.4.1 Equivalence of the relative contribution across the population to the expected relative contribution over a lifetime

Let us consider the expected value of the total contribution that a individual makes to the force of infection over the course of their lifetime, measured in force of infection x time (henceforth, "the total contribution"). We will denote this quantity as Λ. At the endemic equilibrium, the system is stationary. Consequently, at a given point in time, the average contribution of any individual then present in the population to the force of infection (i.e., $\frac{\lambda}{N}$) is simply the ratio of the total contribution to the average duration of a lifetime:

$$\frac{\lambda}{N} = \frac{\Lambda}{L} = \mu\Lambda$$
$$\therefore \lambda = N\mu\Lambda$$

The endemic force of infection is simply the sum of the endemic force of infection from first infections (λ_{First}) and the endemic force of infection from reinfections($\lambda_{\text{Subsequent}}$). The same is true of the total contribution and its components (Λ_{First} and $\Lambda_{\text{Subsequent}}$). Consequently, the logic of the paragraph above applies to these components as well:

$$\lambda_{\text{First}} = N\mu\Lambda_{\text{First}}$$
$$\lambda_{\text{Subsequent}} = N\mu\Lambda_{\text{Subsequent}}$$

And thus we obtain the following equation for the relative contribution of reinfections to the endemic force of infection in terms of the total contributions from first infection and from reinfections:

$$\frac{\lambda_{\text{Subsequent}}}{\lambda_{\text{First}}} = \frac{\Lambda_{\text{Subsequent}}}{\Lambda_{\text{First}}}$$

3.4.2 The total contribution from first infection

The total contribution from first infection is the product of the probability of being infected before dying, for a fully susceptible individual ($\mathbf{P}\left(S \to W_1\right)$); the average duration of a first infection ($\mathbf{D}\left(W_1\right)$); and the contagiousness of a first infection (β), divided – because our model is frequency-

dependent – by the size of the total population (N):

$$\begin{aligned}\Lambda_{\text{First}} &= \mathbf{P}(S \to W_1)\,\mathbf{D}(W_1)\,\beta/N \\ &= \left(\frac{\lambda}{\lambda+\mu}\right)\left(\frac{1}{\gamma+\mu}\right)\beta\left(\frac{1}{N}\right) \\ &= \frac{\lambda\beta}{(\lambda+\mu)(\gamma+\mu)N}\end{aligned}$$

3.4.3 The total contribution from reinfections

The total contribution from reinfections has a similar form, but is slightly more complicated: It is the product of the probability of being reinfected at least once ($\mathbf{P}(S \to W_R)$), the average number of reinfections *given* that one is reinfected at least once ($\mathbf{E}(W_R)$), the average duration of a reinfection ($\mathbf{D}(W_R)$), and the contagiousness of reinfection ($\kappa_c\beta$), divided (as above) by the size of the total population:

$$\Lambda_{\text{Subsequent}} = \mathbf{P}(S \to W_R)\,\mathbf{E}(W_R)\,\mathbf{D}(W_R)\,\kappa_c\beta/N$$

The probability of being reinfected at least once is the product of probability of being infected at all ($\mathbf{P}(S \to W_1)$); the probability of recovering before dying, for a first-infected individual ($\mathbf{P}(W_1 \to R)$); the probability of having one's immunity wane before dying, for a fully-immune individual ($\mathbf{P}(R \to P)$); and the probability of being reinfected before dying, for an individual with waned immunity ($\mathbf{P}(P \to W_R)$):

$$\begin{aligned}\mathbf{P}(S \to W_R) &= \mathbf{P}(S \to W_1)\,\mathbf{P}(W_1 \to R)\,\mathbf{P}(R \to P)\,\mathbf{P}(P \to W_R) \\ &= \left(\frac{\lambda}{\lambda+\mu}\right)\left(\frac{\gamma}{\gamma+\mu}\right)\left(\frac{\omega}{\omega+\mu}\right)\left(\frac{\kappa_s\lambda}{\kappa_s\lambda+\mu}\right) \\ &= \frac{\kappa_s\lambda^2\gamma\omega}{(\lambda+\mu)(\gamma+\mu)(\omega+\mu)(\kappa_s\lambda+\mu)}\end{aligned}$$

Similarly, given that an individual is reinfected at least m times, the probability that they are reinfected at least $m+1$ times ($\mathbf{P}(W_R \to W_R)$) is the product of the probability of recovering before dying, for a reinfected individual ($\mathbf{P}(W_R \to R)$); the probability of having one's immunity wane before dying, for a fully-immune individual; and the probability of being reinfected before dying, for an individual with waned immunity:

$$\begin{aligned}\mathbf{P}(W_R \to W_R) &= \mathbf{P}(W_R \to R)\,\mathbf{P}(R \to P)\,\mathbf{P}(P \to W_R) \\ &= \left(\frac{\kappa_d^{-1}\gamma}{\kappa_d^{-1}\gamma+\mu}\right)\left(\frac{\omega}{\omega+\mu}\right)\left(\frac{\kappa_s\lambda}{\kappa_s\lambda+\mu}\right) \\ &= \frac{\kappa_s\lambda\gamma\omega}{(\gamma+\kappa_d\mu)(\omega+\mu)(\kappa_s\lambda+\mu)}\end{aligned}$$

The expected number of reinfections for an individual who is reinfected at least once is therefore:

$$\begin{aligned}
\mathbf{E}(W_R) &= \sum_{i=0}^{\infty} \mathbf{P}(W_R \to W_R)^i \\
&= \frac{1}{1 - \mathbf{P}(W_R \to W_R)} \\
&= \frac{1}{1 - \frac{\kappa_s \lambda \gamma \omega}{(\gamma + \kappa_d \mu)(\omega + \mu)(\kappa_s \lambda + \mu)}} \\
&= \frac{(\gamma + \kappa_d \mu)(\omega + \mu)(\kappa_s \lambda + \mu)}{(\gamma + \kappa_d \mu)(\omega + \mu)(\kappa_s \lambda + \mu) - \kappa_s \lambda \gamma \omega}
\end{aligned}$$

The average duration of a reinfection is:

$$\begin{aligned}
\mathbf{D}(W_R) &= \frac{1}{\kappa_d^{-1} \gamma + \mu} \\
&= \frac{\kappa_d}{\gamma + \kappa_d \mu}
\end{aligned}$$

And by substituting all of the above results back into the first equation in this subsection, we obtain the following expression for the endemic force of infection from reinfections:

$$\begin{aligned}
\Lambda_{\text{Subsequent}} =& \mathbf{P}(S \to W_R) \mathbf{E}(W_R) \mathbf{D}(W_R) \kappa_c \beta / N \\
=& \left(\frac{\kappa_s \lambda^2 \gamma \omega}{(\lambda + \mu)(\gamma + \mu)(\omega + \mu)(\kappa_s \lambda + \mu)} \right) \left(\frac{(\gamma + \kappa_d \mu)(\omega + \mu)(\kappa_s \lambda + \mu)}{(\gamma + \kappa_d \mu)(\omega + \mu)(\kappa_s \lambda + \mu) - \kappa_s \lambda \gamma \omega} \right) \times \\
& \left(\frac{\kappa_d}{\gamma + \kappa_d \mu} \right) \left(\frac{\kappa_c \beta}{N} \right) \\
=& \left(\frac{\lambda \beta}{(\lambda + \mu)(\gamma + \mu) N} \right) \left(\frac{\kappa_s \kappa_c \kappa_d \lambda \gamma \omega}{(\gamma + \kappa_d \mu)(\omega + \mu)(\kappa_s \lambda + \mu) - \kappa_s \lambda \gamma \omega} \right) \\
=& \Lambda_{\text{First}} \left(\frac{\kappa_s \kappa_c \kappa_d \lambda \gamma \omega}{(\gamma + \kappa_d \mu)(\omega + \mu)(\kappa_s \lambda + \mu) - \kappa_s \lambda \gamma \omega} \right)
\end{aligned}$$

3.4.4 Conclusion

The relative contribution of reinfections to the endemic force of infection is therefore:

$$\begin{aligned}
\frac{\lambda_{\text{Subsequent}}}{\lambda_{\text{First}}} &= \frac{\Lambda_{\text{Subsequent}}}{\Lambda_{\text{First}}} \\
&= \frac{\kappa_s \kappa_c \kappa_d \lambda \gamma \omega}{(\gamma + \kappa_d \mu)(\omega + \mu)(\kappa_s \lambda + \mu) - \kappa_s \lambda \gamma \omega}
\end{aligned}$$

Thus, the relative contribution of reinfections to the endemic force of infection is directly proportional to (and thus, linear in) the relative contagiousness of reinfections (κ_c). It is also positively dependent on both the relative susceptibility to reinfection (κ_s) and the relative duration of reinfection (κ_d), but is sublinear with respect to each of them (Figure 1). However, the degree to which it is sublinear is very different between the two; the dependence on κ_s is strongly sublinear, while the

dependence on κ_d is so close to linear that it cannot be visually distinguished from the truly linear dependence on κ_c. This is because the only place that κ_d appears in the denominator is as part of the term $(\gamma + \kappa_d \mu)$, and $\gamma >> \mu$. Finally, the relative contribution of reinfections to the endemic force of infection is also positively, and sublinearly, dependent on the waning rate (ω) (Figure 2).

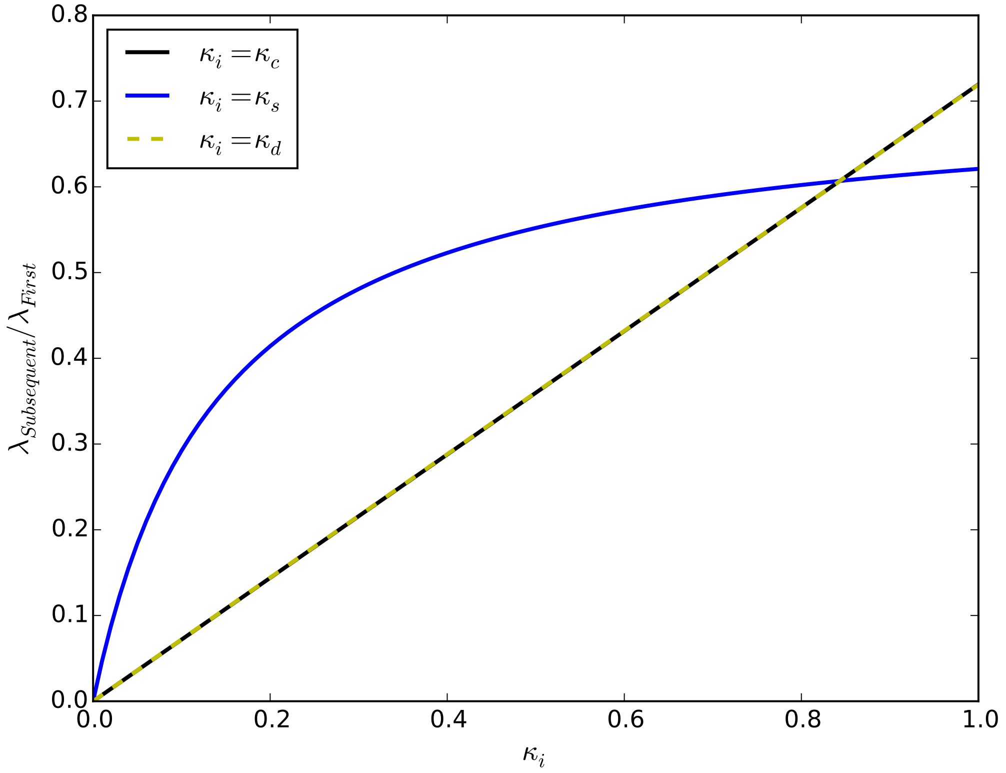

Figure 1: Dependence of $\frac{\lambda_{\text{Subsequent}}}{\lambda_{\text{First}}}$ on κ_c, κ_s, and κ_d. In each curve, all parameters other than the κ_i being varied are set to the values they have in the "slow deep" scenario for the high transmission setting in the main text; in particular, both of the other two κ_j are fixed at $0.6^{1/3}$.

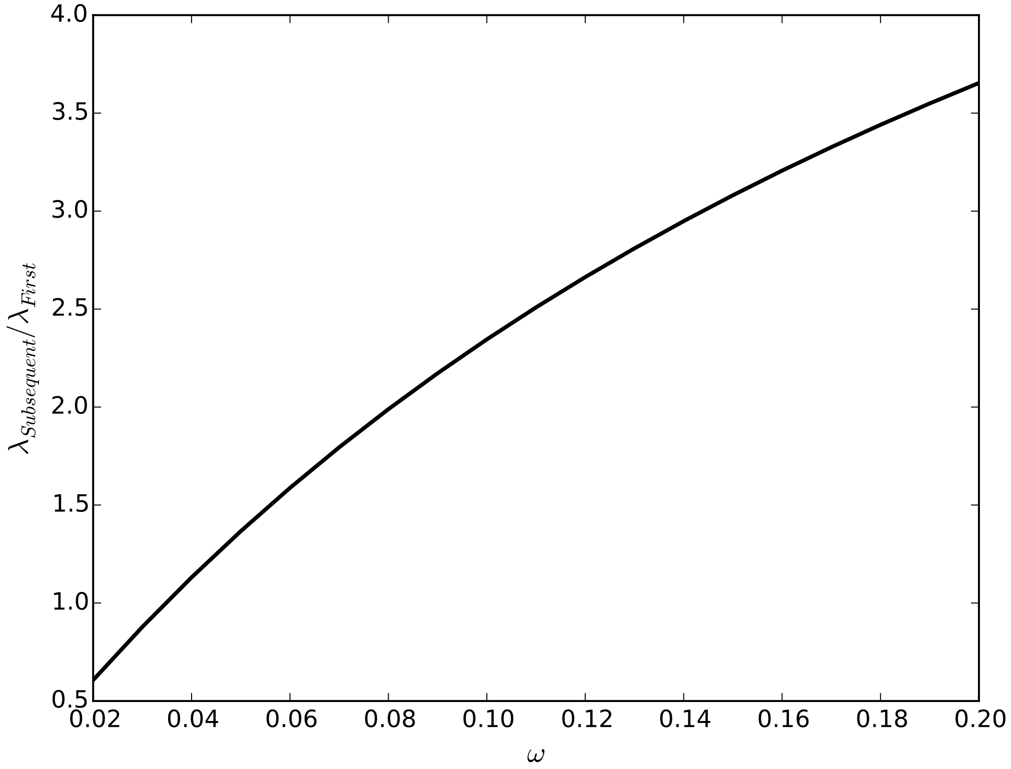

Figure 2: Dependence of $\frac{\lambda_{\text{Subsequent}}}{\lambda_{\text{First}}}$ on ω. All parameters other than ω are set to the values they have in the "slow deep" scenario for the high transmission setting in the main text.

3.5 Simplification when there is only one parameter κ

In our model as actually implemented, $\kappa_s = \kappa_d = \kappa_c = \kappa$. Consequently, we can simplify the above equation slightly:

$$\frac{\lambda_{\text{Subsequent}}}{\lambda_{\text{First}}} = \frac{\kappa^3 \lambda \gamma \omega}{(\gamma + \kappa\mu)(\omega + \mu)(\kappa\lambda + \mu) - \kappa\lambda\gamma\omega}$$

This expression is clearly superlinear in κ, and just as clearly subcubic. But it is not obvious just from glancing at it where in that range it falls. Numerical tests (Figure 3, and others not shown) indicate that it is solidly between quadratic and cubic for the range of parameters considered in any of the scenarios presented in the main text.

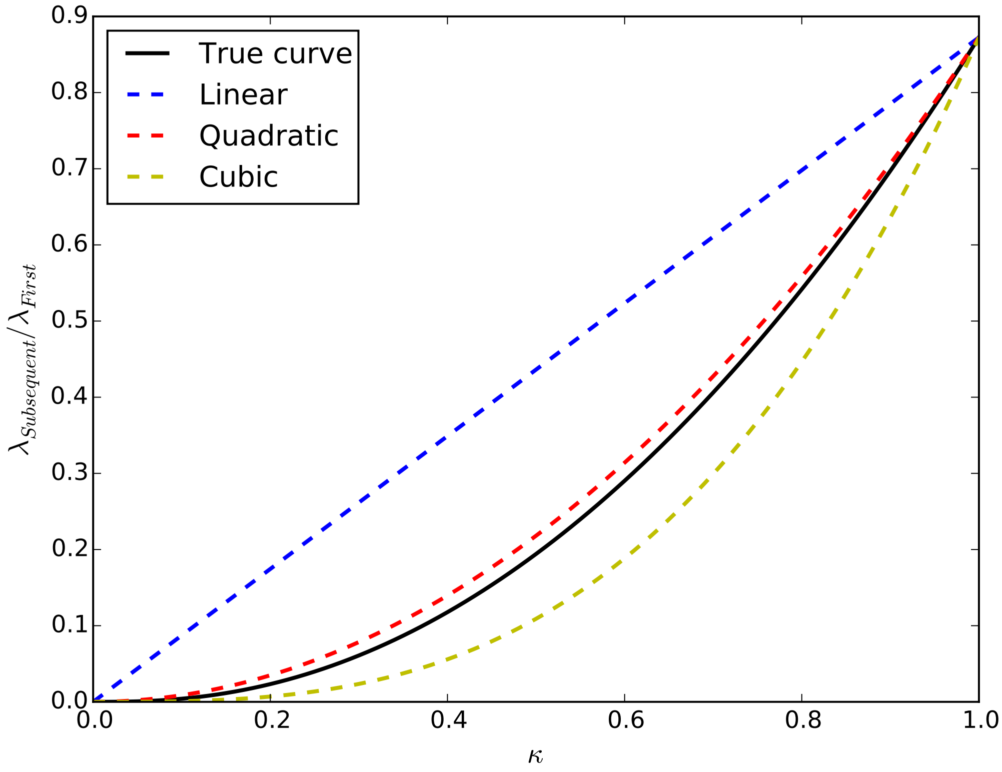

Figure 3: Dependence of $\frac{\lambda_{\text{Subsequent}}}{\lambda_{\text{First}}}$ on κ. All parameters other than κ are set to the values they have in the "slow deep" scenario for the high transmission setting in the main text. For comparison, linear, quadratic, and cubic curves having the same two endpoints are shown as dashed lines.

4 Waning in the Wagner *et al.* (2014) model

4.1 Introduction to the model

One of the more detailed and arguably realistic models of immune boosting and waning following live-virus infection is the one presented by Wagner *et al.* (2014), elaborating on the antibody-based model of Behrend *et al.* (2014). Although a full discussion of that model is beyond the scope of this paper, we will sumarize key aspects of that model, that are relevant to the findings of our model.

4.1.1 Table of parameters

Symbol	Symbol used in Wagner *et al.* (if different)	Value	Meaning
p_s	$\mathrm{P0Inf}_s$	$\begin{cases} 0.17, & s=1 \\ 0.29, & s=2 \\ 0.13, & s=3 \end{cases}$	Infectivity parameter for strain s
D	PVDose_s	[variable]	Dose of oral polio vaccine (of strain s), in TCID_{50}
A_s	$\mathrm{NAb}_{1,s}$	variable	Effective titer of mucosal antibodies to strain s of poliovirus
M_s	$M_{\mathrm{prime},s}$	$\begin{cases} 5.4, & s=1 \\ 6.17, & s=2 \\ 5.51, & s=3 \end{cases}$	Mean logarithm (base 2) of mucosal antibody titer following a live virus infection with strain s in an immunologically naive individual
σ_s	$\sigma_{\mathrm{prime},s}$	$\begin{cases} 2.2, & s=1 \\ 2.5, & s=2 \\ 2.7, & s=3 \end{cases}$	Standard deviation of the logarithm (base 2) of mucosal antibody titer following a live virus infection with strain s in an immunologically naive individual
τ	τ_{Ab}	0.038	"Neutralization factor for poliovirus by antibodies" (Wagner *et al.* 2014)
k_d	k_{DI}	0.0469	Parameter governing reduction in average duration of infection due to partial immunity, in $\log_{10}(\text{days})/\log_2(\text{GMT})$
k_r	k_{shed}	0.0833	Parameter governing reduction in shedding rate due to partial immunity, in $\log_{10}(\text{shed titer})/\log_2(\text{GMT})$ (Wagner 2016)

4.1.2 Probability of infection in the absence of immunity

In the model presented in Wagner *et al.*, the probability of infection for an immunologically naive individual challenged with a dose of D TCID_{50} of oral polio vaccine (OPV) of strain s, before

accounting for strain interference (which is not a feature of our model) is taken to be:

$$1 - \left(1 + \frac{D}{1 - p_s}\right)^{-p_s}$$

where p_s is a strain-specific infectivity parameter.

In a beta-Poisson dose-response model (Furumoto and Mickey 1967), the susceptibility of targets of infection (which may be individuals, cells, or something in between) varies, with the probability of infection when exposed to a single infectious particle following a Beta(α, β) distribution, and each target of infection is exposed to a (Poisson-distributed) average of $D_{\text{particles}}$ infectious particles. Under some further conditions, the probability of infection under these circumstances can be approximated as:

$$1 - \left(1 + \frac{D_{\text{particles}}}{\beta}\right)^{\alpha}$$

Thus, the dose-response model of Wagner *et al.* is an approximate beta-Poisson model, with individuals as the variably susceptible targets of infection, and with the additional constraint that

$$\alpha + \frac{\beta}{c} = 1,$$

where c is the number of infectious particles per TCID_{50} of OPV. This additional constraint reduces the two-parameter (approximate) beta-Poisson model to a one-parameter model. It is not remarked upon or justified in Wagner *et al.*, and is not present in the cited reference (Haas *et al.* 2014). This constraint is clearly carried over from Behrend *et al.* (2014), but is not remarked upon or justified in that paper either.

Presumably, the motivation behind this constraint is that, in the exact beta-Poisson model, $\alpha/\left(\alpha+\beta\right)$ is the mean per-particle probability of infection. Thus, by setting $\beta = 1 - \alpha$, with $\alpha = p_s$, one appears to obtain the result that the mean per-particle probability of infection is p_s, which would be consistent with the symbol (P0Inf_s) used for that parameter in Wagner *et al.*. But in fact, this would only be true if c were 1 (i.e., if each TCID_{50} of OPV consisted of only a single viral particle), and if that were the case, then the low value of β relative to α would render the approximation to the true beta-Poisson model relatively poor. Nevertheless, for the purposes of this appendix, we will simply take this feature of the model as a given.

4.1.3 Acquisition immunity

In the Wagner *et al.* model, immunity to infection is modeled as an unobserved effective titer of mucosal neutralizing antibodies to each of the three serotypes of poliovirus A_s, for $s = 1, 2, 3$. A complete lack of mucosal immunity to serotype s is represented by $A_s = 1$. When an individual is challenged with a dose of D TCID_{50} of OPV of strain s, the probability of infection, taking immunity into account, is:

$$1 - \left(\left(1 + \frac{D}{1 - p_s}\right)^{-p_s}\right)^{\dfrac{1 + \tau\left(A_s - 1\right)\left(1 - e^{-1/\tau}\right)}{A_s}}$$

Note that when $A_s = 1$, the above equation indeed reduces to the simpler equation given in the previous section.

4.1.4 Intensity of shedding

In the model description presented in the supporting information of Wagner *et al.* (2014), the intensity of shedding is implied to be both deterministic (for a given effective mucosal antibody titer) and constant over the duration of infection. However, this contradicts the trajectories of shedding titers over the course of infection shown in Figure 1(B) in the main text of Wagner *et al.* (2014), which are clearly not constant. The authors have indicated (Wagner 2016) that the presentation in the main text is the correct one, and that the dynamics of shedding are those described in the following paragraphs.

In the absence of immunity, the log_{10} of each individual's peak shedding rate ($S_{i,\text{peak}}$) is selected from a trunctated (at $z = \pm 2$) normal distribution; for each $\log_2 (A_s)$, the $\log_{10}$ of $S_{i,\text{peak}}$ is reduced by $k_r = 0.0833$. That individual's shedding rate at a given time since infection ($S_i(t_{inf})$) is then:

$$S_i(t_{inf}) = \begin{cases} 0, & t_{inf} < 1 \\ S_{i,\text{peak}} \left(\dfrac{e^{\frac{-\left(\log\left(t_{inf}\right) - \mu_{LT}\right)^2}{2\sigma_S^2}}}{\left(t_{inf}\right) e^{\frac{\sigma_S^2}{2} - \mu_{LT}}} \right), & 1 \le t_{inf} < T_i \end{cases}$$

where T_i is the duration of that individual's infection, and

$$\sigma_S = 1.8$$
$$\mu_{LT} = 4.65$$

Although the mean and standard deviation of the distribution of peak shedding titers were not specified, a mean of approximately 4.3 $log_{10}(\text{TCID}_{50}/\text{day})$ can be inferred from Figures 1(B) in Wagner *et al.* (2014). In numerical tests (not shown), our results proved highly insensitive both to the precise value of the mean and to even very large changes (several orders of magnitude) in the standard deviation.

4.1.5 Duration of infection

In the absence of immunity, the duration of infection is randomly selected from a normal distribution with a mean of 25 days and an unspecified standard deviation. For each $\log_2 (A_s)$, the mean is reduced by a constant k_d, and the standard deviation is reduced proportionally. In the text of the online Supporting Information of Wagner *et al.*, this reduction is clearly stated to be linear; however, Table S1 of the Supporting Information gives the value of k_d as "0.057 $\log_{10}(\text{days})/\log_2(\text{GMT})$," suggesting that the reduction is proportional. That the reduction is indeed proportional has been confirmed by the authors, who also supplied a corrected value of "0.0469 $\log_{10}(\text{days})/\log_2(\text{GMT})$ (Wagner 2016).

4.1.6 Priming and boosting of immunity

Priming of immunity in an immunologically naive individual and boosting of immunity in an individual with a previous exposure are handled separately. When an individual with $A_s \le 1$ experiences a live virus infection with strain s, the logarithm (base 2) of their post-infection antibody titer is randomly selected from a normal distribution with mean M_s and standard deviation σ_s, which vary from strain to strain.

The Wagner *et al.* model also includes boosting of immunity upon live virus infection of an individual with $A_s > 1$. However, for the purpose of simplicity and conceptual clarity, we only examine waning following a single live virus infection in this appendix. Many of the same results apply if waning after multiple infections is considered; however, specifying the precise assumptions about infection and/or exposure history becomes considerably more complex.

4.1.7 Waning of immunity

Mucosal immunity and humoral immunity are assume to wane separately. However, the model description in the Supplementary Information indicates that only mucosal immunity is treated as relevant to susceptibility to infection, infection duration, and intensity of shedding, with humoral immunity only being relevant (a) to protection from paralysis and (b) as a directly measurable proxy (combined with vaccination history) for the unmeasured mucosal immunity. Therefore, in this appendix, we only consider the waning dynamics of mucosal immunity.

Mucosal antibody levels are assumed to undergo simple exponential decay at a rate of either 0.1/yr. (slow waning) or 0.2/yr. (fast waning).

4.2 Comparison of waning dynamics to those in our model

For illustrative purposes, the rest of this section focuses on the waning dynamics of the Wagner *et al.* model with parameters for Type 3 poliovirus. All of the major qualitative results also hold for the other two serotypes, although the exact numerical values vary.

4.2.1 Acquisition immunity

As seen in figure 4, relative susceptibility to reinfection in our model is comparable to or lower than in the Wagner *et al.* model, in substantial part due to the fact that our model treats individuals who have just recovered from infection as fully immune, while the Wagner model does not. Moreover, the extent of ongoing waning (i.e. continuing waning beyond 5 years after the end of a previous infection) is actually greater in the Wagner *et al.* models than in our models. This suggests that our results regarding the effects of ongoing waning on the potential for sustained silent circulation remain highly relevant when the simplifying assumptions we have made about the nature of waning immunity are relaxed.

4.2.2 Intensity of shedding and average duration of infection

Figure 5 depicts relative average duration of infection in the various models, while Figure 6 covers relative intensity of shedding. These are the same in our model, and are constant over time. This is because, in our model, previously infected individuals are (at any given time) in one of two homogeneous states: Fully immune, or partially susceptible. Fully immune individuals cannot be infected, and partially susceptible individuals all have the same relative duration of infection and shedding rate. Therefore, the expected duration of infection and shedding rate of reinfected individuals does not depend on the time since first infection; the effects of waning immunity come solely from the increase over time in the fraction of the population that is suceptible.

In contrast, in the Wagner *et al.* model, relative susceptibility, relative duration of infection, and relative shedding rate are all dependent on effective mucosal antibody titer, and so effects of waning immunity are seen in all three ratios. This has a somewhat subtle consequence: In order

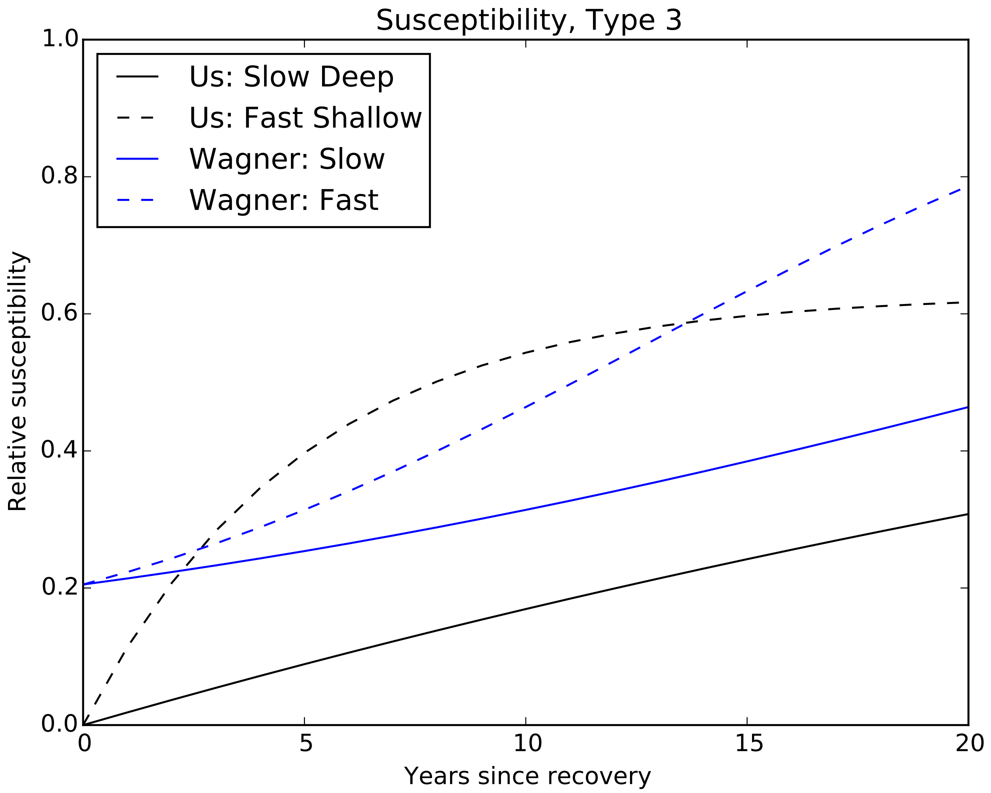

Figure 4: Curves showing relative risk of infection with Type 3 poliovirus (compared to an immunologically naive individual) vs. time since previous Type 3 infection in years, under different models of immune waning. Curves for our model are shown in black, and curves for the Wagner *et al.* model in blue. For each model, the solid line represents the sub-model with slowest waning , while the dashed line represents the sub-model with the fastest waning. The curve for our "intermediate" model is not shown. The curves for the Wagner *et al.* model reflect relative risk of infection following a challenge dose of $10^{5.8}$ $TCID_{50}$ of OPV3; the curves for our model indicate relative risk of infection following a cumulative exposure that would produce the same probability of infection in an immunologically naive individual as $10^{5.8}$ $TCID_{50}$ of OPV3 would produce in the Wagner model.

to appropriately define an average relative duration, it is necessary to weight the distribution of possible antibody titers by the probability of being infected in the first place (given a particular challenge dose). Likewise, in order to appropriately define an average relative shedding rate, it is necessary to weight the distribution of possible antibody titers by the probability of infection times the expected duration. For this reason, the degree to which the curves for relative shedding rate in the Wagner *et al.* model in Figure 6 are higher than the corresponding curves for relative duration in Figure 5 is greater than would be expected from the difference between k_d and k_r alone. This is because individuals who have lower titers have (1) a higher probability of infection, (2) a longer expected duration of infection if infected, and (3) a higher shedding rate during infection. Therefore, weighting by both the probability of infection and the expected duration of infection (as we do when calculating the average relative shedding rate) would result in a higher average than weighting by only the probability of infection (as we do when calculating the average duration of infection), even if the probability distributions were identical apart from this weighting.

As regards both duration of infection and shedding rate, the potential for transmission from reinfections is substantially higher in the Wagner *et al.* model than in ours, although it should be stressed that this observation is made in the context of immunity generated by only a single previous infection, and will naturally be less true the greater the number of previous infections (including vaccinations that succeed in producing a live virus infection). More robust, however, is the observation that ongoing waning is again stronger in the Wagner *et al.* model than in ours.

4.2.3 Overall

In figure 7, we compare the various models with respect to the overall relative potential of previously infected individuals to contribute to transmission, as measure by relative probability of infection times relative average duration of infection times relative shedding rate, analogous to Figure 2 in the main text, which provides that comparison with respect to our models only. Again, we see that both the relative potential for reinfections to contribute to transmission, measured in this way, and the extent to which waning is ongoing are greater in the Wagner *et al.* model than in ours. This strongly suggests that our conclusion that ongoing waning can greatly increase the potential for extended silent circulation is robust to relaxation of our simplifying assumptions about the dynamics of waning of mucosal immunity following live virus infection.

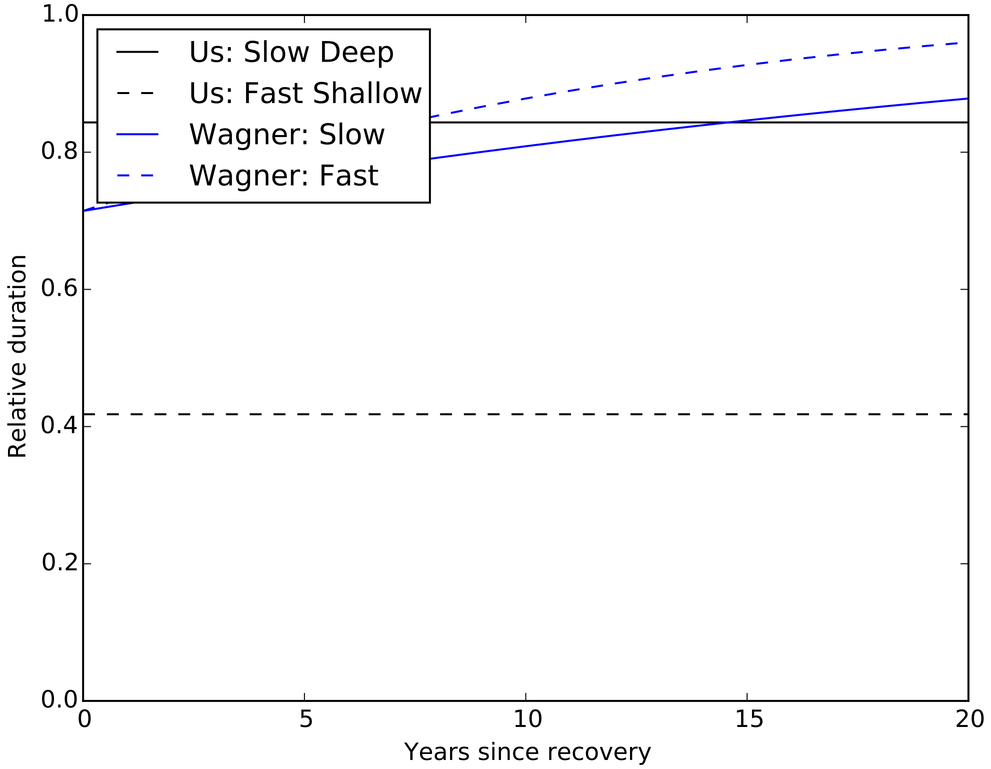

Figure 5: Curves showing relative average duration of infection while infected with Type 3 poliovirus (compared to an immunologically naive individual) vs. time since previous Type 3 infection in years, under different models of immune waning. Curves for our model are shown in black, and curves for the Wagner *et al.* model in blue. For each model, the solid line represents the sub-model with slowest waning , while the dashed line represents the sub-model with the fastest waning. The curve for our "intermediate" model is not shown. The curves for the Wagner *et al.* model reflect relative risk of infection following a challenge dose of $10^{5.8}$ $TCID_{50}$ of OPV3; the curves for our model are the same regardless of challenge dose, for reasons discussed in the text.

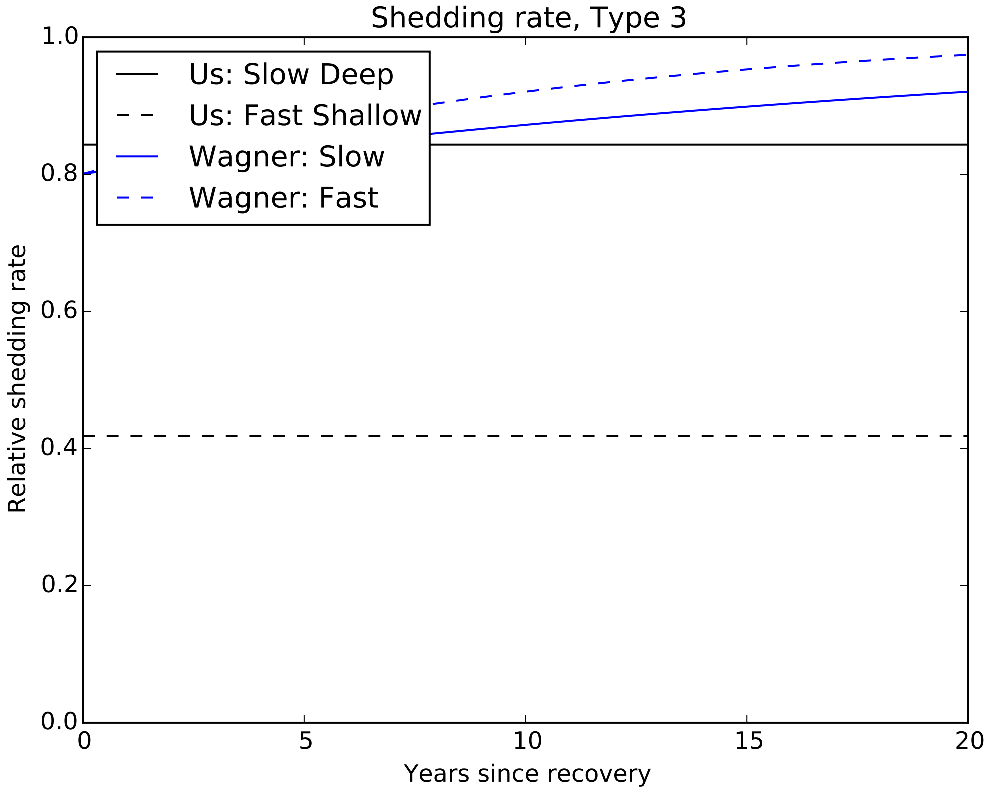

Figure 6: Curves showing relative intensity of virus shedding while infected with Type 3 poliovirus (compared to an immunologically naive individual) vs. time since previous Type 3 infection in years, under different models of immune waning. Curves for our model are shown in black, and curves for the Wagner *et al.* model in blue. For each model, the solid line represents the sub-model with slowest waning , while the dashed line represents the sub-model with the fastest waning. The curve for our "intermediate" model is not shown. The curves for the Wagner *et al.* model reflect relative risk of infection following a challenge dose of $10^{5.8}$ $TCID_{50}$ of OPV3; the curves for our model are the same regardless of challenge dose, for reasons discussed in the text.

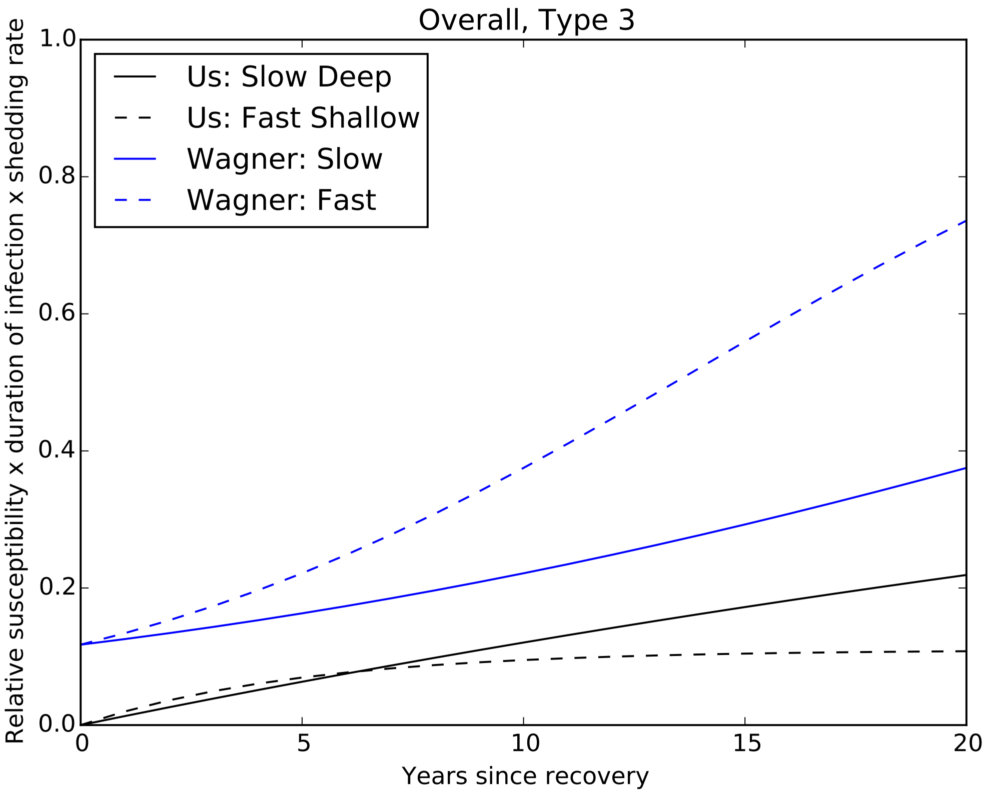

Figure 7: Curves showing relative probability of infection with Type 3 poliovirus times relative average duration of infection times relative shedding rate (compared to an immunologically naive individual) vs. time since previous Type 3 infection in years, under different models of immune waning. For our model, this is equivalent to the "population extent of waning" shown in Figure 2 in the main text. Curves for our model are shown in black, and curves for the Wagner *et al.* model in blue. For each model, the solid line represents the sub-model with slowest waning , while the dashed line represents the sub-model with the fastest waning. The curve for our "intermediate" model is not shown. The curves for the Wagner *et al.* model reflect relative risk of infection following a challenge dose of $10^{5.8}$ $TCID_{50}$ of OPV3; the curves for our model are the same regardless of challenge dose, for reasons discussed in the text.

5 Effect of varying vaccination levels at the end of a 20 year ramp up

Here we examine an additional aspect of vaccination history that affects the risk of prolonged silent circulation. That is the prevalence of first infections at the end of a slow ramp up of vaccination. We varied this by varying the end point vaccination levels as described in Table 1.

In Figure 8 we present final vaccination level sweeps for silent circulation duration like those seen in Figure 4 in the main text, but restricted to the 20 year ramp up delay, the higher level of transmission, and fast shallow or intermediate waning scenarios. The difference between fast shallow waning and intermediate waning is remarkable. That difference is due to the same phenomenon that produced the differences in Figure 7 in the main text. Fast shallow waning does not have much ongoing waning after 5-10 years and therefore reinfections do not continue to displace the contribution of first infections to the effective reproduction number as time goes on. Therefore the rate of vaccinations required to eliminate the last polio case does not rise as it does with intermediate level waning.

For intermediate waning, however, changing the endpoint prevalence of first infections makes a big difference. Figure 9 illustrates the two reasons for this, both of which result from the fact that a lower prevalence of first infections requires a higher vaccination rate (before the jump) to achieve. First, at a lower prevalence of first infections (and hence, a higher pre-jump vaccination level), there is a lowernumber of completely susceptible children under five. This is seen as a decreasing height in the <5 completely susceptible (green) at the end of the ramp up as the prevalence of first infections at the end of the ramp up is lowered. As a boost in vaccination rates primarily reduces the contribution to the basic reproduction number of children under five, this means that a given jump in vaccination rates will produce a smaller reduction in the basic reproduction number. Second, because the vaccination level before the jump will be higher, the size of the boost needed to achieve a given total vaccination rate after the jump is smaller. This leads to a lower fractional decrease in the height of the <5 year old contribution to the effective reproduction number after the boost in vaccination levels. Thus, a weaker boost combines with a weaker effect for a given strength of boost to produce a much smaller effect overall.

Note that at the final prevalence level at the end of the ramp up of 50 in Figure 9, the last polio case is seen before the end of the vaccination ramp up. In some cases that means that the jump in the vaccination level at the end of the ramp up is negative. That makes it more difficult to interpret what is going on in Figures like Figure 4 in the main text and makes those Figures harder to interpret. Even at the final total WPV infection level of 100 at the end of the vaccination ramp up, we saw some parameter settings where this occurred. That is why we chose 300 as the end of ramp up total WPV prevalence in the main text.

Table 1: Vaccination levels at the end of 20 year ramp-ups needed to achieve specified first infection prevalence levels at the end of the ramp up by waning scenario

End Point Prevalence	Fast Shallow End Point Vaccination Levels	Intermediate End Point Vaccination Levels
300	0.33	0.42
100	0.45	0.66
50	0.50	0.81

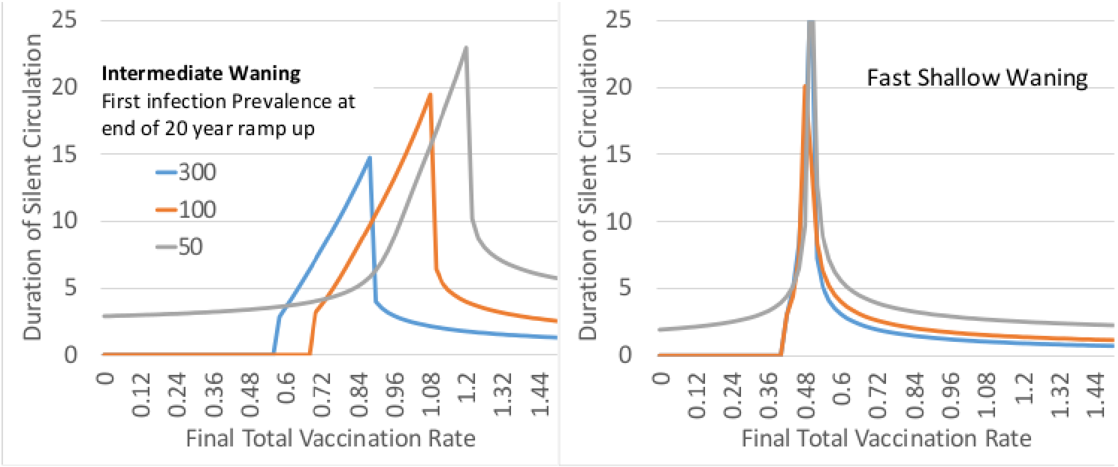

Figure 8: Silent circulation duration as a function of final total vaccination levels across values the endpoint prevalence of first infection from 300 to 50. Settings are for the intermediate and fast shallow waning scenario, a 20 year ramp-up time, and poliovirus type 3 infection to paralysis ratios and intermediate transmissibility of vaccine.

But the distortion just mentioned should not cause us to miss the important observation that if there is a slow approach to very low levels of WPV first infections that allows the total effective reproduction number to stay close to one due to the slow compensatory dynamics of more transmissions occurring from reinfections, then those compensatory dynamics will make prolonged silent circulation more likely. Those compensatory dynamics decrease the contribution of the less than five age group of unvaccinated individuals to the effective reproduction number. That means that it takes even higher levels of final vaccination rates in less than five year olds to get enough of a drop in the effective reproduction number to lead to eradication. In the real world it is quite possible that the step by step improvements in vaccination coverage that eventually lead to elimination of polio cases could be getting prevalence to very low levels before the last polio case is seen. In the presence of any ongoing waning in older individuals, the analyses for Figures 8 and 9 show that would raise the duration of silent circulation above what is presented in Figure 4 of the main text.

Consideration of these dynamics leads us to speculate that in places like Nigeria and Pakistan, the risk of prolonged silent circulation will be high. It might be further increased by adding further realism to our model. Our model has homogeneous mixing. Given homogeneous mixing it may take a rather precise history of vaccination to slowly approach a very low level of vaccination that eliminates polio cases while still sustaining poliovirus circulation. But we hypothesize that a more realistic real world model would have reintroductions of infection to areas that eradicated transmission locally through adequate vaccination levels. Such reintroductions could be from neighboring areas with lower levels of vaccination that occur after a further period of waning that enhances the potential of reinfections to sustain circulation. Such added realism would increase the chances of sustaining prolonged low level silent circulation. Note that if there is fine grained variation of this sort, this could markedly decrease the size of a population needed to sustain circulation of polioviruses.

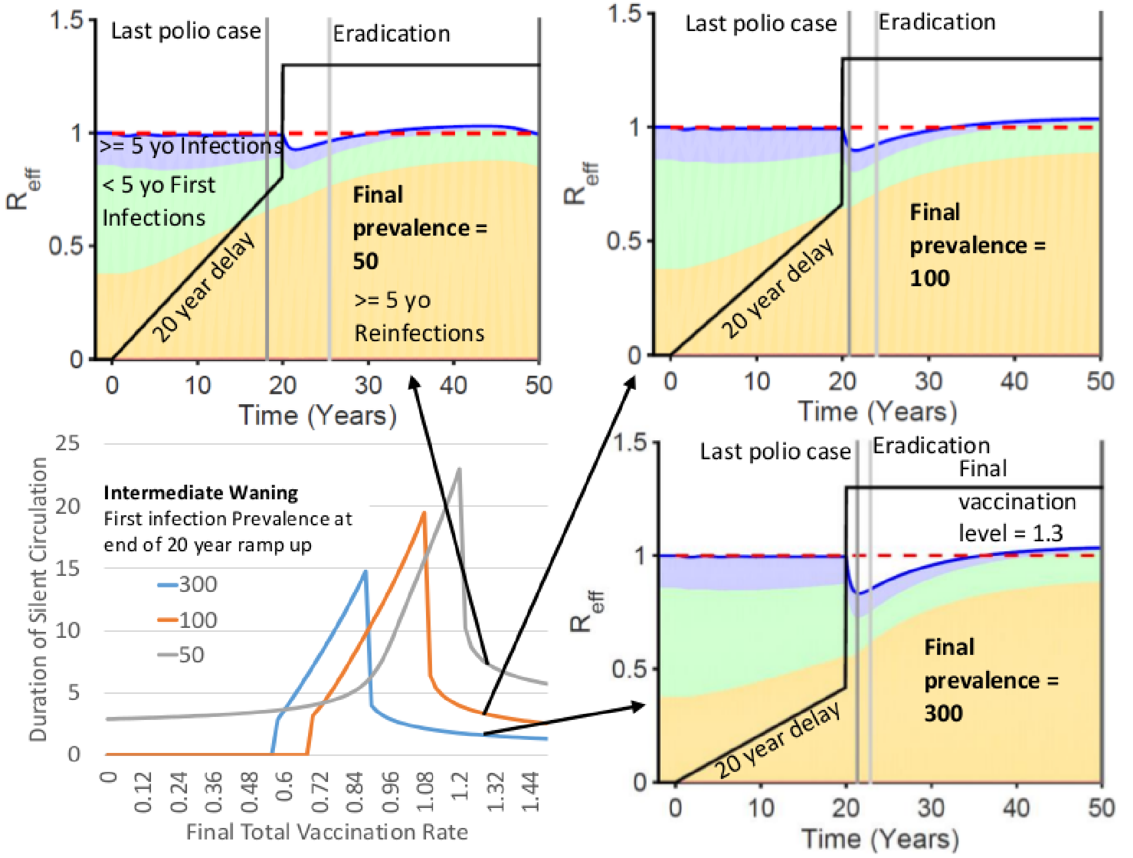

Figure 9: Comparison of effective reproduction number dynamics as the first infection prevalence at the end of a 20 year ramp up becomes increasingly lower for intermediate waning.

6 Works cited

1. Behrend, M.R., Hu, H., Nigmatulina, K.R., & Eckhoff, P. A quantitative survey of the literature on poliovirus infection and immunity. *Int J Infect Dis* **18**, 4-13. (2014)

2. Diekmann, O., Heesterbeek, J. A. P. & Roberts, M. G. The construction of next-generation matrices for compartmental epidemic models. *J. R. Soc. Interface* **7**, 873885 (2010).

3. Dietz, K. The estimation of the basic reproduction number for infectious diseases. *Stat. Methods in Med. Res.* **2**, 23-41 (1993)

4. Furumoto, W.A. & Mickey R. A mathematical model for the infectivity-dilution curve of tobacco mosaic virus: theoretical considerations. *Virology.* **32**(2) (1967)

5. Haas CN, Rose JB, & Gerba CP Quantitative Microbial Risk Assessment. Wiley. (2014)

6. Wagner BG. Personal communication. (2016)

7. Wagner BG, Behrend MR, Klein DJ, Upfill-Brown AM, Eckhoff PA, & Hu H. Quantifying the impact of expanded age group campaigns for polio eradication. *PLoS One.* **9**(12) (2014) doi: 10.1371/journal.pone.0113538
